# Neural adaptation accounts for the dynamic resizing of peripersonal space: evidence from a psychophysical-computational approach

**DOI:** 10.1152/jn.00652.2017

**Published:** 2018-03-14

**Authors:** Jean-Paul Noel, Olaf Blanke, Elisa Magosso, Andrea Serino

**Affiliations:** ^1^Laboratory of Cognitive Neuroscience, Brain Mind Institute, Faculty of Life Science, Ecole Polytechnique Federale de Lausanne, Lausanne, Switzerland; ^2^Center for Neuroprosthetics, Ecole Polytechnique Federale de Lausanne, Lausanne, Switzerland; ^3^Vanderbilt Brain Institute, Vanderbilt University, Nashville, Tennessee; ^4^Department of Neurology, University of Geneva, Geneva, Switzerland; ^5^Department of Electrical, Electronic, and Information Engineering “Guglielmo Marconi,” University of Bologna, Cesena, Italy; ^6^MySpace Lab, Department of Clinical Neuroscience, Centre Hospitalier Universitaire Vaudois, University of Lausanne, Lausanne, Switzerland

**Keywords:** multisensory, neural adaptation, neural network model, peripersonal space, plasticity, receptive fields

## Abstract

Interactions between the body and the environment occur within the peripersonal space (PPS), the space immediately surrounding the body. The PPS is encoded by multisensory (audio-tactile, visual-tactile) neurons that possess receptive fields (RFs) anchored on the body and restricted in depth. The extension in depth of PPS neurons’ RFs has been documented to change dynamically as a function of the velocity of incoming stimuli, but the underlying neural mechanisms are still unknown. Here, by integrating a psychophysical approach with neural network modeling, we propose a mechanistic explanation behind this inherent dynamic property of PPS. We psychophysically mapped the size of participant’s peri-face and peri-trunk space as a function of the velocity of task-irrelevant approaching auditory stimuli. Findings indicated that the peri-trunk space was larger than the peri-face space, and, importantly, as for the neurophysiological delineation of RFs, both of these representations enlarged as the velocity of incoming sound increased. We propose a neural network model to mechanistically interpret these findings: the network includes reciprocal connections between unisensory areas and higher order multisensory neurons, and it implements neural adaptation to persistent stimulation as a mechanism sensitive to stimulus velocity. The network was capable of replicating the behavioral observations of PPS size remapping and relates behavioral proxies of PPS size to neurophysiological measures of multisensory neurons’ RF size. We propose that a biologically plausible neural adaptation mechanism embedded within the network encoding for PPS can be responsible for the dynamic alterations in PPS size as a function of the velocity of incoming stimuli.

**NEW & NOTEWORTHY** Interactions between body and environment occur within the peripersonal space (PPS). PPS neurons are highly dynamic, adapting online as a function of body-object interactions. The mechanistic underpinning PPS dynamic properties are unexplained. We demonstrate with a psychophysical approach that PPS enlarges as incoming stimulus velocity increases, efficiently preventing contacts with faster approaching objects. We present a neurocomputational model of multisensory PPS implementing neural adaptation to persistent stimulation to propose a neurophysiological mechanism underlying this effect.

## INTRODUCTION

Virtually all interactions between an agent and the environment are mediated by the body and occur within the peripersonal space (PPS), that is, the space immediately adjacent to and surrounding the body (di Pellegrino et al. 1997; Rizzolatti et al. 1981, 1997). The ecological relevance of the near space is evidenced in that the primate brain has developed a frontoparietal network dedicated to the representation of PPS (Graziano et al. 1997; Graziano and Cooke 2006; Grivaz et al. 2017). Neurons within this network are multisensory (Avillac et al. 2007; Bernasconi et al. 2018), in that they respond to tactile stimulation on the body, and also to visual (Duhamel et al. 1997, 1998; Schlack et al. 2005) or auditory stimuli (Graziano et al. 1999) presented close to, but not far from, the body. Furthermore, these multisensory neurons possess depth-restricted receptive fields (RF) that are body part specific and anchored on this body part (Duhamel et al. 1998; Graziano et al. 1997, 1999, see Brozzoli et al. 2011, 2012a for a similar observation in humans). Adaptively, these RFs change in size both plastically and dynamically, properties further revealing the significance of the PPS system in avoidance and defensive behavior, as well as primate-world interactions.

It has been demonstrated that following prolonged utilization of a tool enlarging the reach of action, the size of PPS neurons’ RF enlarges plastically as to incorporate the tool (Iriki et al. 1996 in monkeys and Farnè and Làdavas 2000 in humans). This phenomenon of PPS resizing over the course of numerous repetitions and experimental factors is well established both in monkeys (Maravita and Iriki 2004) and in humans (Bassolino et al. 2010; Galli et al. 2015; Noel et al. 2015a, 2015b, 2017; Salomon et al. 2017; Serino et al. 2007) and has arguably been the primary area of psychophysical study within the field. Furthermore, neural network models (see Magosso et al. 2010b, Serino et al. 2015a) with recurrent feedforward/feedback connections between spatiotopically organized unisensory and multisensory areas have proposed a putative computational framework based on Hebbian learning (Hebb 1949) accounting for the plasticity of PPS representation over repeated trials.

On the other hand, PPS has been also shown to adapt dynamically, i.e., instantaneously, as a function of the nature of sensory-motor interaction between the individual and the environment (Cléry et al. 2015a, 2015b). For instance, PPS enlarges toward the goal of an action the subject is planning or executing (Brozzoli et al. 2009, 2010; Noel et al. 2015b). Moreover, in a seminal paper, Fogassi et al. (1996) demonstrated that, as the velocity of incoming visual stimuli increased, the size of the RFs of multisensory neurons also increased, as if to initiate the computation for PPS representation earlier and integrate the speed of the incoming stimuli (see Colby et al. 1993 for a similar finding and Bremmer et al. 2013 for further neurophysiological evidence describing dedicated intraparietal neurons encoding for movement in the near extrapersonal space). This property is categorically different from the plasticity property described above, as it does not require repeated exposures but acts online as a function of the current stimulation. The dynamism of PPS representation fits well with a major role ascribed to this space, i.e., that of predicting in an online fashion possible collisions with stimuli and preparing a potential motor response (Cléry et al. 2015a, 2017; Graziano and Cooke 2006). Differently from the plasticity case, nonetheless, it is unknown whether the dynamic properties of monkey PPS equally apply to humans, and there is no data in humans showing a velocity-dependent resizing of PPS. Furthermore, there is no neurophysiological and biologically plausible computational framework able to account for dynamic remapping of PPS (but see Straka and Hoffmann 2017, for an artificial intelligence approach to the modeling of PPS’ dynamism).

Here, we attempt to close this gap by a combination of psychophysical experimentation and neural network modeling. First, we show in humans, via an audio-tactile psychophysical task (Canzoneri et al. 2012; Serino et al. 2015a, 2018), that the representation of both peri-face and peri-trunk space enlarged when approaching stimuli increased in velocity. Subsequently, we used a neural network model to formalize a possible neural mechanism underlying these psychophysical results. To this aim, we adapt a network we previously developed to describe the representation of the peri-hand space (Magosso et al. 2010a, 2010b; Serino et al. 2015a) maintaining unchanged its architecture and the majority of its parameters (see methods), to reproduce the multisensory properties of PPS neurons mapping both the peri-trunk and peri-face space (Serino et al. 2015b). Briefly, each PPS network is formed by layers of unimodal areas (tactile and auditory in this case to reproduce the audio-tactile stimulation used in the corresponding behavioral experiments), which project to a multisensory area, integrating inputs from the different senses. The strength of the synapses from unisensory to multisensory neurons are arranged so that unisensory neurons with receptive fields on or near the body send inputs strong enough to activate the multisensory neurons when a stimulus is within their receptive fields, whereas neurons with far receptive fields send weak signals to multisensory neurons not inducing a multisensory response. In this manner the model reproduces the basic properties of PPS neurons, i.e., mapping in a multisensory fashion the space near the body. Then, we used the model to propose and test a neurophysiological mechanism accounting for the enlargement of both these PPS representations when stimuli approach the body with higher speeds. Specifically, the network assumes a neural adaptation mechanism to persistent stimulation as the key machinery for dynamic PPS resizing (see Graziano and Cooke 2006 for a discussion of neural adaptation within the frontoparietal PPS network). More precisely, in the neurocomputational model, we implemented a progressive reduction in the evoked activity of neurons over prolonged stimulation. That is, slower (and therefore, longer) stimuli produce a greater reduction in a neurons’ responsiveness than faster (and therefore shorter) stimuli do (i.e., neural adaptation). Indeed, neural adaptation is a pervasive phenomenon across the different sensory systems (Barlow and Mollon 1982) and, interestingly, has been documented to closely reflect the natural statistics of the world (Webster and Mollon 1997) in a similar fashion to multisensory processes (Parise et al. 2014; Stein and Meredith 1993). In fact, Elliott et al. (2009) proposed that neural adaptation at unisensory areas shapes the neural input to multisensory areas in such a manner to optimize the emergence of multisensory enhancement.

Conceptually, the effect of adaptation within a neural network can be described as follows; when stimuli are slow as opposed to fast, a particular neuron within a spatiotopically organized neural ensemble is activated for a longer interval of time and thus is subject to neural adaptation. This adaptation weakens the contribution of the particular neuron to the networks’ activity and globally decreases the output of the network itself. In turn, reaction times to audio-tactile stimuli are modified as a function of the velocity of incoming auditory stimuli, and network properties engender a resizing of PPS. The implementation of this mechanism into our PPS neural network supposes the transition from a descriptive to a mechanistic model and was able to reproduce behavioral results collected here. Furthermore, the reproduction of behavioral data is shown to depend on the neural architecture being robust against parameter variations and equally is generalizable to account for neurophysiological data reported by Fogassi et al. (1996).

## METHODS

### Participants

Thirty-two subjects (11 women, mean age = 24.1 ± 1.5) partook in this study. Participants were divided in two groups: those around which peri-face representation was mapped (16 subjects, 5 women) and those around which peri-trunk representation were mapped (16 subjects, 6 women). Data from 1 subject in the peri-trunk group was excluded due to technical problems. All participants were right handed, had correct or corrected-to-normal visual acuity, and reported normal hearing and touch. All participants gave written, informed consent to take part in this study, which was approved by the local ethics committee, The Brain Mind Institute Ethics Committee for Human Behavioral Research at Ecole Polytechnique Federale de Lausanne. All participants were remunerated with 20 Swiss Francs for their time.

### Stimulus and Apparatus

Participants were outfitted with a vibrotactile device (Precision MicroDrives shaftless vibration motors, model 312-101, 3V, 60 mA, 150 Hz, 5 g) to deliver tactile stimulation. The motor had a surface area of 113 mm^2^ and reached maximal rotation speed in 50 ms. This device was placed either on the participant’s forehead (peri-face group) or on the chest, at sternum level (peri-trunk group) and was activated for 100 ms during tactile stimulation. The acoustic stimuli consisted of dynamic broadband noise looming toward participants at either 25 cm/s (Slow) or 75 cm/s (Fast) traveling for 2 m at a constant loudness of 50 dB. The audio rendering system was composed of two uniform linear arrays of eight loudspeakers each (JBL Control 1 Pro WH Pair, M-Audio FastTrack Ultra 8R). These arrays were placed alongside participants (50 cm in each direction in the horizontal plane) and extended distally (in front) for 2 m maintaining altitude. For each participant individually, the loudspeakers were placed either at the altitude of their face (peri-face group) or their chest (peri-trunk group). The algorithm governing sound movement has been extensively explained elsewhere (Serino et al. 2015b) and is therefore omitted here. Lastly, participants were blindfolded and handed a wireless gamepad (XBOX 360 controller, Microsoft, Redmond, WA, 215 Hz sampling rate), which they held in their right hand and used to respond to vibrotactile stimulation.

### Procedure

Participants were informed that they would feel a tactile vibration and hear a sound approaching toward them. They were informed that the sound was task irrelevant and that their task was to respond as accurately and rapidly as possible to tactile vibration by pressing a button on the gamepad. They were also informed that in some trials (baseline trials) only tactile vibration would be administered and on other trials (catch trials) only the sound would be presented.

The study consisted of a between-subjects variable (peri-face and peri-trunk mapping) and two within-subjects variables: *1*) Sound Velocity (Slow vs. Fast), and *2*) Sound Distance (D1 through D7). Mapping of PPS around the face and trunk were identical with exception of the placement of the vibrotactile device and the altitude of the array of speakers. At the onset of a trial, either a Slow (25 cm/s) or Fast (75 cm/s) sound loomed toward the participant. Then, at a certain interval from sound onset (Slow: T1 = 1 s, T2 = 2 s, T3 = 3 s, T4 = 4 s, T5 = 5 s, T6 = 6 s, T7 = 7 s; Fast: T1 = 0.33 s, T2 = 0.66 s, T3 = 1.00 s, T4 = 1.33 s, T5 = 1.66 s, T6 = 2.00 s, T7 = 2.33 s), the vibrotactile stimulation was given. The different temporal intervals of tactile stimulation were selected so that the broadband noise was perceived at a given different distance from the body. The correspondence between the temporal interval from sound onset and the spatial distance between sound and touch location match linearly and negatively (T1 through T7 are, respectively, equivalent to D7, D6, D5, D4, D3, D2, and D1). In all cases D1 = 25 cm, D2 = 50 cm, D3 = 75 cm, D4 = 100 cm, D5 = 125 cm, D6 = 150 cm, and D7 = 175 cm. In addition to the fact that as looming sounds are delayed they are by default physically closer to participants, in a prior study utilizing the exact same setup we have demonstrated that perceptually there is no deviance from linearity. That is, participants perceive linearly approaching sounds as linearly approaching (see Fig. 1 in Serino et al. 2015b).

**Fig. 1.**
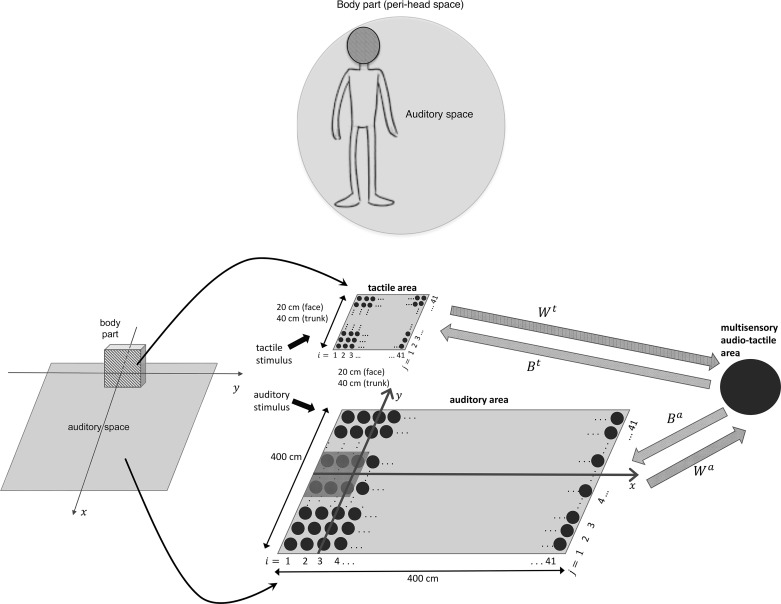
The neural network architecture. This same architecture holds for both the peri-face space network and peri-trunk space network. Each network includes a unisensory tactile area, coding the skin on the frontal surface of the body part, a unisensory auditory area representing the space extending laterally and in front of the body part, and a multisensory audio-tactile area. Each filled circle represents a neuron. Larger cycles mean neurons having larger receptive fields. *W^t^* and *W^a^* denote the feedforward synapses from the unisensory tactile and auditory neurons, respectively, to the multisensory neuron. *B^t^* and *B^a^* are the feedback synapses from the multisensory neuron to the unisensory tactile and auditory neurons, respectively. The small darker region in the auditory area marked the space on the body part [20 cm along *x* and 40 cm along *y* (20 cm on each side) in case of the trunk and 20 cm along *y* (10 cm on each side) in case of the face]. The auditory neurons code the auditory space in body part-centered coordinates (*x*, *y*), having origin on the frontal surface of the body part.

In addition to these experimental trials, baseline and catch trials were inserted within the randomization. Baseline trials were unimodal tactile trials given at the temporal equivalents of D1 and D7. These trials were compared with experimental audio-tactile trials to demonstrate a facilitation effect on tactile processing due to auditory stimulation close to the body, as opposed to a putative nonspecific expectancy or anticipation effect (see *Behavioral Analysis*). Lastly, catch trials were trials in which auditory stimuli were presented yet no vibrotactile stimulation was given, and therefore participants were to withhold response. This last condition was included to avoid an automatic association between the sounds and a motor response. All trial types were randomized, and each condition was repeated 16 times for a total of 320 trials per participant and experiment [2 (Slow vs. Fast) × 10 (D1 through D7 + (D1 + D7 baselines) + catch) × 16 repetitions].

### Behavioral Analysis

Reaction times (RTs) to vibrotactile stimulation were analyzed. In a first step, RTs that were faster than 200 ms or slower than 900 ms were discarded. Subsequently, data for each condition was amalgamated independently, and RTs above or below 2.5 SD of the mean of its condition were rejected. For each subject individually their median for each condition was taken, as RT distributions are generally skewed and the median is less affected by the presence of outliers (Whelan 2008). Next, for each subject individually and for each sound velocity condition, we subtract the participant’s tactile unimodal fastest condition (D1 or D7) from the median RTs in all experimental trials. That is, we correct experimental RTs (e.g., reaction times to audio-tactile stimuli) by the fastest baseline unimodal RTs, hence adopting the most conservative approach possible to categorize multisensory enhancement. By definition, thus, unimodal baseline is equal to zero, and negative values indicate facilitation in RT in the multimodal condition as compared with the mentioned unimodal baseline.

When the distribution of median RTs for all participants was not normally distributed as assessed via Mauchly’s Test of Sphericity we performed nonparametric statistics. For instances when no appropriate nonparametric test exists (e.g., 3-way mixed model ANOVA) we conducted standard analyses of variances, correcting for sphericity via the Greenhouse-Geisser correction. First we conducted a 2 [(body part: Face vs. Trunk) × 2 (velocity: 25 cm/s vs. 75 cm/s) × 7 (Sound Distance: D1 through D7)] mixed model ANOVA. Then, as results indicate a 3-way interaction, two separate (for each body part) 2 (fast sound vs. slow sound) × 7 (audio-tactile distances) repeated-measures ANOVAs are conducted to test for a distance effect, e.g., audio-tactile RTs modulated by the spatial discrepancy between the two unisensory components that is dependent on sound velocity (i.e., a sound velocity × distance interaction). Subsequently, for each sound velocity and PPS measure (e.g., face and trunk), we carry out one-sample *t*-tests against a null distribution centered on zero (data were normally distributed) to ascertain the location of the PPS boundary, operationally defined as the furthest location in distance at which audio stimuli enhance tactile processing.

Once the presence of a PPS effect was confirmed, to more fine-grain delineate the extent of the different PPS representations as a function of the velocity of the incoming sound, we fit multisensory RTs to a sigmoidal function according to *Eq. 1*

(1) $$y\left(x\right)=\frac{y_{min}+y_{max}\times e^{\left(x-x_{c}\right)/b}}{1+e^{\left(x-x_{c}\right)/b}}$$

*x* represents the distance (in cm) between auditory and tactile stimuli, and *y*(*x*) is the RT to touch at a given auditory distance *x*. *y_min_* and *y_max_* are saturation points of the sigmoidal fixed to the slowest and fastest median RT in the experimental trials, while *x_c_* and *b*, respectively, represent the central point and the slope of the sigmoidal at *x_c_* and are free to vary. The central point of this function is taken as a behavioral proxy for the size of PPS: the location of the PPS boundary (see Canzoneri et al. 2012; Serino et al. 2015a, for a similar approach). The estimated values of the sigmoidal central point were compared first via mixed-models ANOVA and then either via planned paired (comparison of velocities) or unpaired *t*-tests (comparison of peri-face vs. peri-trunk space).

### Neural Network Description

Two neural networks were used to simulate the peri-face space and peri-trunk space. The two networks share the same architecture and the vast majority of parameter values, inherited from our previous neural networks describing the visuo-tactile and audio-tactile interaction in the peri-hand space (Magosso et al. 2010a, 2010b; Serino et al. 2015a). That is, for exception of the adaptation mechanism, the neural network model implemented here is identical to that previously reported for the peri-hand space (Serino et al. 2015a) with only a few parameters modified to account for differences in PPS size depending on body parts, as suggested by neurophysiology (see *Peri-Face and Peri-Trunk Space* below). In the following, we first provide a general description of the neural network architecture; then, the novel aspects compared with our previous networks are emphasized. Only a few key mathematical equations are reported. All other network equations are the same as in our previous works (Magosso et al. 2010a, 2010b; Serino et al. 2015a). Table 1 lists all network parameters.

**Table 1.** Values of peri-face space network and peri-trunk space network

Unisensory neurons’ receptive fields (amplitude ${\text{Φ}}_{0}$ and standard deviation ${\text{σ}}_{\phi }$ of the bidimensional Gaussian function)			
${\text{Φ}}_{0}^{t}$ = 1	${\text{σ}}_{\text{Φ}}^{t}$ = 0.5 cm	${\text{Φ}}_{0}^{a}$ = 1	${\text{σ}}_{\text{Φ}}^{a}$ = 10 cm

External tactile and auditory stimuli (amplitude S and standard deviation ${\text{σ}}_{I}$ of the bidimensional Gaussian function)			
$S^{t}$ = [3.3–3.7]*	$\sigma_{I}^{t}$ = 0.3 cm	$S^{a}$ = [6–8]*	$\sigma_{I}^{a}$ = 6 cm

Lateral synapses in the unisensory areas (amplitude L and standard deviation σ of the bidimensional excitatory and inhibitory Gaussian functions)			
$L_{ex}^{t}$ = 0.75	$L_{in}^{t}$ = 0.25	$\sigma_{ex}^{t}$ = 1 cm	$\sigma_{in}^{t}$ = 4 cm
$L_{ex}^{a}$ = 0.75	$L_{in}^{a}$ = 0.25	$\sigma_{ex}^{a}$ = 20 cm	$\sigma_{in}^{a}$ = 80 cm

Feedforward and feedback synapses (*Eqs. 5–7*)			
${\text{W}}_{0}^{t}$ = 6.5	${\text{W}}_{0}^{a}$ = 6.5	${\text{B}}_{0}^{t}$ = 2.5	${\text{B}}_{0}^{a}$ = 2.5
$k_{1}$ = 40 cm	$k_{2}$ = 700 cm	α = 0.9	
$X_{C}^{F}$ = [−20 ÷ 0] cm	$Y_{C}^{F}$ = [−10 ÷ 10] cm	$X_{C}^{T}$ = [−20 ÷ 25] cm	$Y_{C}^{T}$ = [−20 ÷ 20] cm

Dynamics, sigmoidal activation function, and adaptation mechanism of unisensory neurons (*Eqs. 2–4*)			
$f_{min}$ = −0.12†	$f_{max}$ = 1	r = 0.34	
$\vartheta_{0}$ = 12	G = 0.08	τ = 40 ms	T = 600 ms

Dynamics, sigmoidal activation function, and adaptation mechanism of the multisensory neuron (*Eqs. 2–4*)			
$f_{min}^{m}$ = 0	$f_{max}^{m}$ = 1	$r^{m}$ = 1	
$\vartheta_{0}^{m}$ = 13	$G^{m}$ = 0.005	$\tau^{m}=\tau$ = 40 ms	$T=T^{m}$ = 600 ms

* Ranges of the uniform distribution the strengths of the tactile stimulus and auditory stimulus were drawn from, to simulate tactile RTs in multisensory condition (Figs. 9 and 10). The values used for the strength of the stimuli in the other simulations are specified in each figure legend.

† Since parameter $f_{min}$ had a negative value for the unisensory neurons, a Heaviside function was introduced to avoid that unisensory neurons’ activity became negative (Magosso et al. 2010a, 2010b; Serino et al. 2015a).

Each network includes two areas of unisensory neurons (tactile and auditory, respectively) and a third area of multisensory audio-tactile neurons (Fig. 1). We make use of nonspiking neurons (i.e., the output of each neuron is a continuous variable representing the neuron’s firing rate) that are reciprocally connected by synaptic weights. Each neuron responds to its overall input via a first-order temporal dynamics (mimicking the postsynaptic membrane time constant) and a sigmoidal transfer function (mimicking the neuron’s activation function), generating an output that represents the neuronal firing rate. In the following, we will refer to the neuron’s output as neuron’s activity or neuron’s response.

Each unisensory area is composed by a matrix of *N* × *N* (*N* = 41) unisensory neurons. Unisensory neurons have their own RF, with a bidimensional Gaussian shape, through which the incoming stimulus is filtered, and are topologically aligned (i.e., proximal neurons respond to proximal spatial stimuli). The tactile unisensory neurons respond to tactile stimuli on the frontal surface of the body part. Their RFs are arranged at a distance of 0.5 cm along each dimension for the face and at a distance of 1 cm along each dimension for the trunk, thus mapping a surface of 20 cm × 20 cm (i.e., roughly the face surface) and 40 cm × 40 cm (i.e., roughly the trunk surface), respectively, and reproducing the different spatial acuity of face and trunk regions (Weinstein 1968). The auditory unisensory neurons respond to auditory inputs in a planar space that extends sideways and in front of each body part; their RFs are in body part centered coordinates and are arranged at a distance of 10 cm along each dimension, mapping a space of 400 cm × 400 cm. Moreover, unisensory neurons within each area are reciprocally connected via lateral synapses having a Mexican-hat pattern (near excitation and far inhibition). The tactile area in the network may represent unimodal somatosensory regions in the parietal lobe. The auditory area in the network mimics high-tier stages along the auditory pathway that encode the spatial location of the sound source (Graziano et al. 1999); this piece of information could be extracted from lower-tier auditory stages (and thus not modeled here) based on monaural and binaural cues (Blauert 1983; Recanzone and Sutter 2008). In addition, we assumed that the inputs impacting on the auditory area are already in body part centered coordinates such as in Graziano et al. 1999, and hence we avoid modeling the transformation of sound location among different reference frames (from head-centered coordinates to face- or trunk-centered coordinates).

Neurons in the two unisensory areas send excitatory feedforward synapses (*W*) to the downstream audio-tactile area. This area mimics multisensory regions in the frontoparietal cortex (e.g., ventral premotor cortex, ventral intraparietal area, area 7b), devoted to the representation of body part specific PPS (e.g., peri-face, peri-trunk; see Grivaz et al. 2017 for a review). As electrophysiological data stress the existence of multisensory neurons having large receptive fields covering an entire body part, for parsimony only one multisensory neuron is included in each network (see Magosso et al. 2010b and Serino et al. 2015a, for a similar approach). The feedforward synapses from the tactile neurons to the multisensory one have a uniform value (i.e., their value is independent from the RF’s position of the tactile neurons). In this way, the multisensory neuron has a tactile RF covering the whole body part. The strength of the feedforward synapses from the auditory neurons depends on the distance of the auditory neurons’ RF from the body part. These synapses assume a maximum value for auditory neurons coding for the space bordering the body part, then their value decreases exponentially as the distance of the auditory neurons’ RF from the body part increases. This way the multisensory neuron responds to auditory stimuli within a limited portion of the space around the body part. Finally, the multisensory neuron sends excitatory feedback synapses (*B*) to the tactile and auditory unisensory neurons; the feedback synapses have the same pattern as the feedforward ones.

According to the previous description, the overall input to the unisensory neurons is made up of the external input coming from outside the network (i.e., the stimulus filtered by the neurons’ RFs), plus the lateral input coming from other neurons in the same area, plus the feedback input from the multisensory neuron. The overall input to the multisensory neuron is made up of the feedforward inputs from the two unisensory areas. Within this architecture, in case of a multimodal stimulation, the influence that the auditory stimulus may exert on the tactile activation is mediated via the multisensory area, through the feedforward and feedback synapses. When the auditory stimulus does not trigger the multisensory neuron (i.e., the stimulus activates a region in the unisensory area having null or mild feedforward synapses), the tactile activation is unaffected by the auditory stimulus. When the auditory stimulus is able to trigger the multisensory neuron (i.e., the stimulus activates a region in the unisensory area having strong feedforward synapses), the tactile activity is enhanced via the feedback synapses. Indeed, in our previous reports (Magosso et al. 2010a, 2010b; Serino et al. 2015a), this architecture was shown to reproduce properties of peri-hand space, such as facilitated tactile detection and reduced tactile reaction time by auditory (or visual) stimuli administered near (but nor far from) the hand.

Here, we introduce the following novel aspects to our modeling work.

#### Network stimuli.

In our previous simulation studies, we implemented only static tactile and auditory (or visual) stimuli, i.e., stimuli located at a fixed position on the skin or in space, and maintained constant throughout the entire simulation. This simplification cannot be adopted when the direction and velocity of the auditory stimulus are to be taken into account. Therefore, here we implemented stimuli that reproduce more faithfully the real ones: dynamic auditory stimuli looming toward the body part at different velocities, starting from a distance of 200 cm, and transient tactile stimuli. Both the tactile and auditory stimuli are mimicked by bidimensional Gaussian functions with small standard deviations (i.e., high precision in space) to simulate localized stimuli (see Table 1 for specific parameter value). The tactile and auditory stimuli are convolved with the tactile and auditory neurons’ RF respectively, to generate the input effectively impacting on each neuron—the external input. It is worth noting that each external stimulus (either tactile or auditory) produces not only the activation of the neuron at the central position, but also the surrounding neurons whose RFs cover that position. That is, an ensemble of activated neurons emerges in the corresponding unisensory area. Of course, in case of a moving auditory stimulus, the ensemble of activation shifts along the auditory area as the stimulus shifts.

To simulate a looming sound, the central position (*x_p_*, *y_p_*) of the corresponding Gaussian function is moved along the coordinate *x*, while it is kept fixed along the coordinate *y*, at *y_p_* = 0 cm, so that the sound moves along the central trajectory (see Fig. 1). The simulation starts at *t* = 0 ms with the sound at position (*x*_0_) corresponding to 200 cm distance from the body part, then *x_p_* is updated at every time step Δ*t* = 1 ms as *x_p_* = *x*_0_ −Δ*t*·*vel*/1,000, where *vel* is the arranged velocity (cm/s). The simulation ends when the sound reaches the frontal surface of the body part located at *x* = 0 cm (e.g., the simulation ends at *t* = 8,000 ms if *vel* = 25 cm/s and at *t* = 2,640 if *vel* = 75 cm/s). The amplitude of the Gaussian function (*S^a^*) sets the strength of the stimulus and is maintained constant throughout the entire simulation. It is important to note that, as slower sounds take a longer time to cover a given distance, an auditory neuron at a given position along the sound path receives external input that lasts longer in case of slower than faster sounds.

A transient tactile stimulus is mimicked by a Gaussian function, having central position fixed in a given location of the skin, and amplitude (*S^t^*) different from 0 only at a given temporal interval during which it is maintained constant. This allows applying a tactile stimulus when the sound is at a given distance from the body part, in turn replicating the audio-tactile trials used in the experimental tasks (see *Simulation of the experimental tasks*).

#### Neuronal adaptation mechanism.

We implemented a mechanism to reproduce the phenomenon of adaptation in sensory processing, i.e., the change in a sensory neuron’s responsiveness induced by its recent stimulation history (Barlow and Mollon 1982; Mountcastle et al. 1966, Talbot et al. 1968; Webster and Mollon 1997). Among the repertoires of this phenomenon, one effect is the reduction in the response gain of a sensory neuron when exposed to unchanging stimuli, e.g., long-lasting or repeated stimulation. To simulate adaptation, we operated on the neuron’s activation function. In particular, as briefly explained above, the overall input [say *u*(*t*)] to a generic neuron in the network is processed via a first-order temporal dynamics (*Eq. 2*) and a sigmoidal activation function (*Eq. 3*), generating the neuron’s output activity [say *z*(*t*)]:

(2) $$\tau \frac{\text{d}q\left(t\right)}{\text{d}t}=-q\left(t\right)+u\left(t\right)$$

(3) $$z\left(t\right)=\frac{f_{min}+f_{max}\cdot exp\left\{\left[q\left(t\right)-\vartheta \left(t\right)\right]\cdot r\right\}}{1+exp\left\{\left[q\left(t\right)-\vartheta \left(t\right)\right]\cdot r\right\}}$$

*Equation 2* describes the first-order dynamics, where *q*(*t*) is the state variable and τ the time constant. *Equation 3* describes the sigmoidal activation function; *f_min_* and *f_max_* represent the lower and upper saturation of the sigmoidal function, ϑ establishes the central value of the sigmoidal function (i.e., the input value at which the output is midway between *f_min_* and *f_max_*), and *r* defines the slope. It is worth noting that the sigmoidal activation functions are constrained between 0 and 1. Thus, neuron’s output activity *z*(*t*) assumes values in the range between 0 and 1, where 0 indicates a silent neuron and 1 indicates a maximally activated neuron. *f_min_*, *f_max_*, and *r* in *Eq. 3* are constant parameters. On the contrary, the central value of the sigmoidal function is not maintained constant (as in our previous works, Magosso et al. 2010a, 2010b; Serino et al. 2015a), but it has been assumed to change dynamically on the basis of the previous history of the neuron’s activity, according to the following equation:

(4) $$\vartheta \left(t\right)=\vartheta_{0}+G\cdot \overset{t}{\underset{t-T}{\int }}z\left(\xi \right)d\xi$$

*Equation 4* effectively implements the process of neural adaptation, where *T* is the width of the time window over which the neuron’s previous activity influences the current one, *G* is a positive factor that establishes the amount of adaptation, and $\vartheta_{0}$ is the basal central value of the sigmoidal function, i.e., in case of null neuron’s activity in the previous time window *T*. In the numerical implementation of the model, the ordinary differential equation *Eq. 2* is solved via the forward Euler method with integration time step Δ*t* = 1 ms, and the integral in *Eq. 4* is computed with the histogram rule (with Δ*t* = 1 ms). The higher the value of ϑ, the larger the input required to activate the neuron at the same output level. Therefore, according to *Eq. 4*, as a neuron responds to a stimulus, ϑ(t) progressively increases above $\vartheta_{0}$ and the gain of subsequent responses decreases. Conversely, as the neuron returns to its silent state, after a delay necessary for the previous response to exit from the adaptation window, ϑ(t) progressively declines back toward $\vartheta_{0}$ and the gain of subsequent responses increases.

In the network, in accord with biological observations (Bruno et al. 2010; Heron et al. 2013) the adaptation mechanism mainly takes place in the unisensory areas; for simplicity, the same parameter values (*G* and *T*; see Table 1 for their basal/default values, but also results for a sensitivity analysis on these parameters) have been adopted for neurons both in the tactile area and in the auditory area. On the contrary, we assumed a negligible adaptation mechanism in the multisensory area (parameter *G* for the multisensory neurons, say *G^m^*, was set at a much smaller value than for unisensory neurons; see Table 1 for its default value, but also results for a sensitivity analysis on this parameter). This choice is justified to not violate the principle of multisensory integration according to which two bimodal stimuli presented sequentially (within 100–250 ms) produce enhancement and not depression of the multisensory neuron’s response (Meredith et al. 1987; Stein and Meredith 1993) and is in accord with studies suggesting that neural adaptation precedes (Bruno et al. 2010; Heron et al. 2013) and benefits (Elliott et al. 2009) multisensory integration.

**Fig. 2.**
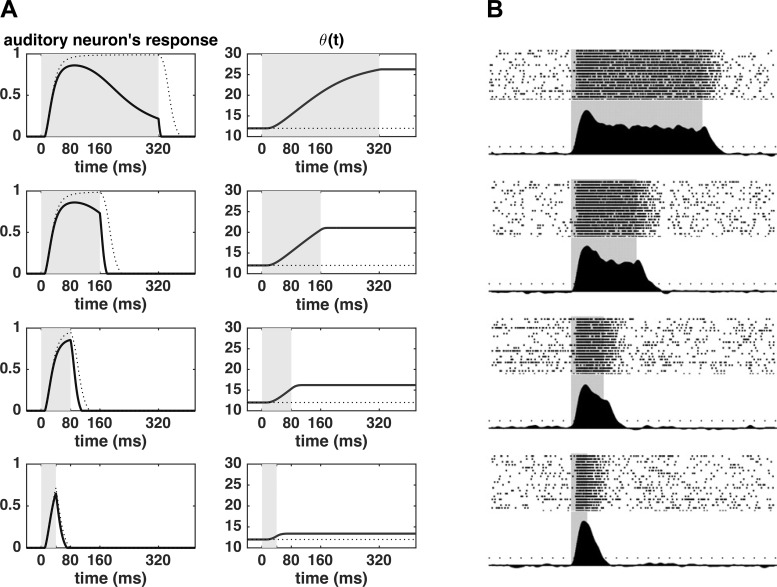
*A*: simulated response (*left*) and pattern of sigmoidal function ϑ(t) (*right*) of an exemplary auditory neuron to stimuli of different durations (320, 160, 80, 40 ms, from *top* to *bottom*) when the adaptation mechanism is operating (thick black lines) and when it is not operating (thin dashed lines). Shaded areas indicate stimulus duration. The strength of the stimulus was *S^a^* = 3.5, and the stimulus was centered in the position coded by the neuron. To isolate the effect of adaptation, the feedback from the multisensory neuron to the auditory ones was nulled in these simulations. *B*: recorded response of a auditory neuron to stimuli of the same duration (320, 160, 80, 40 ms, from *top* to *bottom*), redrawn from Qin et al. (2009) with permission.

Figure 2*A* displays the effect of the adaptation mechanism in an exemplary neuron of the auditory area in the network. The auditory neuron’s response to stimuli of different duration (320, 160, 80, 40 ms) is presented, when the adaptation mechanism is operating, together with the temporal pattern of ϑ(t). For comparison, the corresponding patterns when the adaptation mechanism is not operating are shown too. Some considerations can be drawn. First, despite adaptation, the duration of neuron’s response matches stimulus duration. Long-lasting stimuli produce stronger adaptation, i.e., larger increase in ϑ(t). Note that in all cases ϑ(t) increases almost linearly during the neuron’s response, then it retains the acquired value as long as the previous neuron’s response is within the adaptation window. As a consequence, during long-lasting stimulation, the neuron’s response exhibits a progressive decline after the initial increase (see in particular the case of 320-ms stimulation and to a lesser extent the case of 160-ms stimulation). In case of stimuli of shorter duration, the adaptation effect is almost negligible. Overall, the simulated patterns of the auditory neuron’s response are in line with those obtained by single unit recordings in auditory areas (an exemplary recording in A1 is reported in Fig. 2*B* for the same stimuli durations; Qin et al. 2009). The duration of neuron’s response is correlated with the duration of the stimulus and the responses clearly exhibit a decline after the initial peak during longer stimulation.

**Fig. 3.**
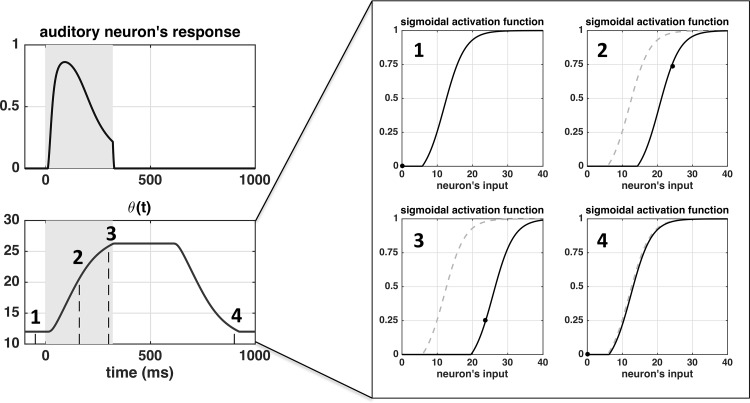
Shift in the sigmoidal activation function of the auditory neurons induced by the dynamic changes in the quantity ϑ(t), during a 320-ms-long stimulation (same simulation as in the top plots of Fig. 2*A*). Four time instants were considered with respect to the stimulus onset (0 ms): −50, 160, 300, 900 ms. The pattern of the sigmoidal function ϑ at −50 ms (*inset 1*) corresponds to the basal one (i.e., $\vartheta =\vartheta_{0}$) and it is reported in dashed gray line in the other *insets* too, for comparison. The full black circle in each *inset* shows the working point of the neuron on the corresponding activation function.

To further clarify how the adaptation mechanism operates, Fig. 3 shows the modification in the neuron’s sigmoidal activation function induced by the dynamic changes in ϑ(t) in case of a 320-ms stimulation (i.e., the same simulation as in the top plots of Fig. 2*A*). During the stimulation, the increase in ϑ(t) causes the activation function to shift toward the right, decreasing the gain of neuron’s response. As the auditory neuron returns to its silent state and the previous response moves out from the adaptation window, ϑ(t) decreases to its basal value and the activation function shifts back to the left, increasing the gain of neuron response.

**Fig. 4.**
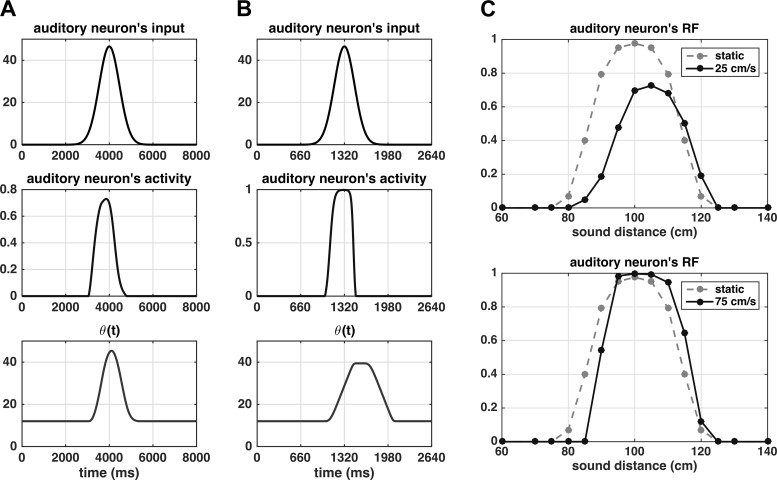
*A* and *B*: time pattern of the external input (*top*), neuron’s activity (*middle*), and quantity sigmoidal function ϑ (*bottom*) for the auditory neuron coding position 100 cm along the sound path, in the case of sounds moving at 25 cm/s (*A*) and 75 cm/s (*B*). The position coded by the neuron is reached at time *t* = 4,000 ms by the sound moving at 25 cm/s and at time *t* = 1,320 ms by the sound moving at 75 cm/s. To account only for the effect of the stimulus velocity on the neuron’s activity, the feedback synapses from the multisensory neuron were nullified. The strength of the stimulus was $S^{a}$ = 7. *C*: the same responses of the auditory neurons (lines and markers) are reported as a function of sound position (neuron responses are sampled at discrete times corresponding to a given sound position), to obtain spatial neuron’s reaction time (RF) to moving sounds. For comparison, each plot also shows the neuron’s RF in response to static stimuli (gray shaded line and marker); this was obtained by computing the maximum response of the neuron to a 200-ms static stimulus (*S^a^* = 4.5) applied in different positions. Slower sounds produce higher levels of neuron fatigue.

**Fig. 5.**
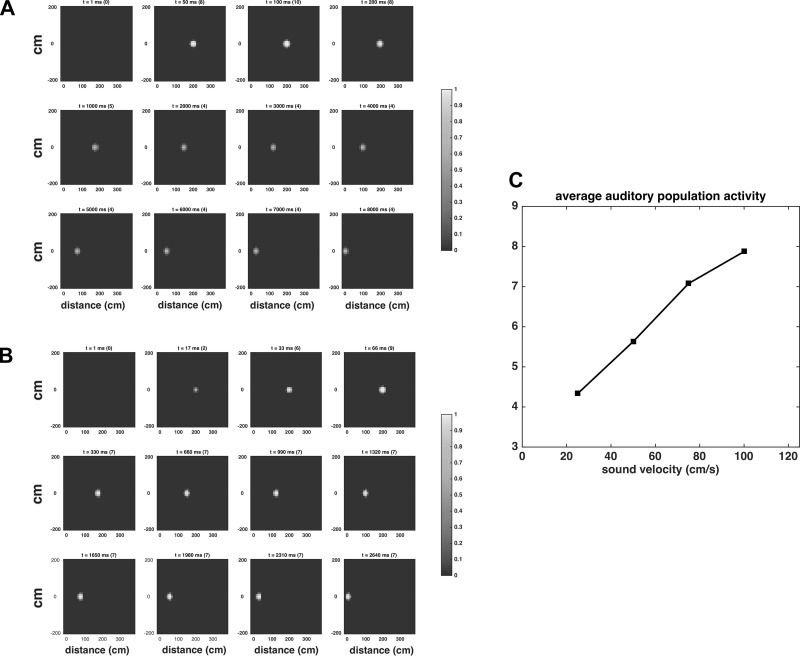
*A* and *B: s*napshots at different time instants, representing the activity of each neuron in the auditory area when simulating a looming sound at 25 and 75 cm/s, respectively. In both cases, the strength of the external auditory stimulus was the same (*S^a^* = 7), i.e., the two simulations differ only for the stimulus velocity. The feedback from the multisensory neuron to the auditory neurons were nullified to account only for the effect of the stimulus velocity. The number within parentheses above each color map indicates the overall auditory activity (i.e., the sum of all auditory neurons’ activity) at the corresponding time instant *t*. Since neuron’s activity is normalized to 1 (maximum activity), a higher number means a larger number of neurons at higher levels of activity. After an initial transient, the faster sound produces a higher level of auditory activity. *C*: the mean overall activity in the auditory area elicited by a moving sound is reported against sound velocity.

It is important to observe that, while the same adaptation mechanism has been implemented both in the auditory and tactile areas, in the present study adaptation to tactile stimulation does not play a relevant role. Indeed, in agreement with the stimuli used in vivo here we simulated only 100-ms tactile stimulation; as we showed previously (Fig. 2*A*), stimuli of such duration would lead to a negligible adaptation effect. Conversely, we argue that the adaptation mechanism may play an important role in the auditory area and be implicated in the adaptive changes of PPS size as a function of incoming stimuli velocities. Indeed, a sound moving at a lower speed would remain over the receptive field of a neuron for a longer period of time, thus being equivalent to a long-lasting unchanging stimulus; conversely, a fast-moving stimulus crosses rapidly the neuron’s RF. Therefore slow- and fast-moving sounds may engender different levels of adaptation in auditory neurons. This is illustrated in Fig. 4, which represents the input to an exemplary auditory neuron (the one coding for a distance of 100 cm), the neuron’s response, and the changes in the quantity ϑ of the neuron’s sigmoidal function as a function of time, in case of sound velocity of 25 cm/s (Fig. 4*A*) and 75 cm/s (Fig. 4*B*). Note the different time scale in the two panels. To improve the comparison, Fig. 4*C* displays the same neuron’s responses (at the two sound velocities) as a function of sound position (specifically, responses were sampled at discrete time instants corresponding to given sound positions with respect to preferred neuron’s position). The input to the neuron progressively increases as the sound approaches the position coded by the neuron, peaks when the sound reaches that position, and then declines as the sound moves away from the position coded by the particular neuron. This input pattern develops over a longer time interval in the case of a slow sound in comparison to a fast sound (Fig. 4, *A* and *B*, *top*). The temporal pattern of the input due to sound motion has important consequences. First, in both cases, the network predicts that the auditory neuron RF shifts toward the approaching sound: i.e., the neuron’s responses are no longer symmetrical around the neuron preferred position; rather, the neuron shows greater responses to the sound entering the RF than exiting it (Fig. 4*C*). This is the consequence of neuron adaptation that develops progressively while the sound crosses the neuron’s RF. It must be noted that this is a novel prediction, and one that is not neurophysiologically tested here but may be of interest in subsequent experiments (see also discussion). Second, and particularly relevant here, a slower sound that requires more time to cross the RF produces a higher level of adaptation, with a consequent stronger reduction gain in neuron’s responsiveness (impeding the neuron to responds maximally even when the sound reaches its preferred position). At the level of the population of auditory neurons, the global effect is that a faster moving sound produces an overall higher activation in the auditory area (i.e., the activation of a larger number of neurons at a higher level of activation) than a slower moving sound (see Fig. 5, *A* and *B*, respectively, for the slow and fast sound). Hence, the velocity of the moving sound is encoded by the population of auditory neurons (faster velocities are coded by higher population activities, see Fig. 5*C*). This, of course, will affect the overall input that the multisensory neuron receives from the auditory area via the feedforward synapses and can shape the auditory RF of the multisensory neuron as a function of sound velocity (see results).

#### Peri-face and peri-trunk space network.

We implemented two networks, devoted to peri-face and peri-trunk space representation, respectively. Neurophysiological data indicate that multisensory neurons related to PPS representation have visual or auditory RFs that extend from the body surface proportionally to the size of the corresponding tactile RF; that is, the larger the size of the body part covered by the tactile RF, the larger the extension of the visual or auditory RF (see Blanke et al. 2015, for a review). In particular, most multisensory neurons in the ventral intraparietal area or ventral premotor cortex have tactile RFs on the face, and visual/auditory RF extending ~10–60 cm from it. Multisensory neurons in area 7b mostly have tactile RFs on the trunk and visual/auditory RFs extending over larger regions of space that may reach even 1 m in depth (Leinonen et al. 1979, Leinonen and Nyman 1979). To account for the difference in depth restriction of auditory representation around the different body parts, the two networks are characterized by different parameter values of the auditory feedforward and feedback synapses (also different from the peri-hand representation in Serino et al. 2015a).

In both the peri-face space and peri-trunk space network, we adopted the same functions as in our previous models (Magosso et al. 2010b, Serino et al. 2015a) to compute the auditory synapses. With reference to a generic auditory neuron *ij* in the map (*i* = 1, 2, …, 41; *j* = 1, 2, ..., 41), having the RF centered at coordinates $x_{i}^{a}=i\cdot 10-30$ (cm) and $y_{j}^{a}=j\cdot 10-210$ (cm) (see Fig. 1), the auditory feedforward (*W*) and feedback (*B*) synapses are given by:

(5) $$W_{ij}^{a,P}=\alpha \cdot W_{0}^{a}\cdot exp\left(-\frac{D_{ij}^{P}}{k_{1}}\right)+\left(1-\alpha \right)\cdot W_{0}^{a}\cdot exp\left(-\frac{D_{ij}^{P}}{k_{2}}\right)P=F,T$$

(6) $$B_{ij}^{a,P}=\alpha \cdot B_{0}^{a}\cdot exp\left(-\frac{D_{ij}^{P}}{k_{1}}\right)+\left(1-\alpha \right)\cdot B_{0}^{a}\cdot exp\left(-\frac{D_{ij}^{P}}{k_{2}}\right)\;P=F,T$$

*P* = *F* refers to the face and *P* = *T* to the trunk. *D_ij_* is a function of the coordinates of the auditory neuron’s RF, computed as

(7) $$D_{ij}^{P}=min\sqrt{{\left(x_{i}^{a}-X_{C}^{P}\right)}^{2}+{\left(y_{j}^{a}-Y_{C}^{P}\right)}^{2}}\;P=F,T$$

*X_C_* and *Y_C_* define a set of coordinates in the *x* and *y* direction, respectively, so that *R* = *X_C_*∪*Y_C_* identifies a region in the auditory space on and near the body part. According to the previous equations, *D_ij_* is null for the auditory neurons having the RF centered within the region *R*, and the feedforward and feedback synapses of these neurons assume the maximum value $W_{0}^{a}$ and $B_{0}^{a}$. For the auditory neurons having the RF outside the region *R*, *D_ij_* computes the minimum Euclidean distance of the RF center from this region, and the synapses decrease as *D_ij_* increases, according to a biexponential function (*Eqs. 5* and *6*). In *Eqs. 5* and *6*, *k*_1_ and *k*_2_ are the fast and slow decay rate, respectively, and α sets the relative amplitude of each exponential. We define the region *R* larger for the trunk than for the face, so that synapses start to decrease at larger distances from the trunk than from the face. This way we account for the different size of auditory PPS representation for the two body parts in a parsimonious way, keeping all other parameters in *Eqs. 5* and *6* at the same value (see Table 1 for default values of parameters defining feedforward and feedback connections).

**Fig. 6.**
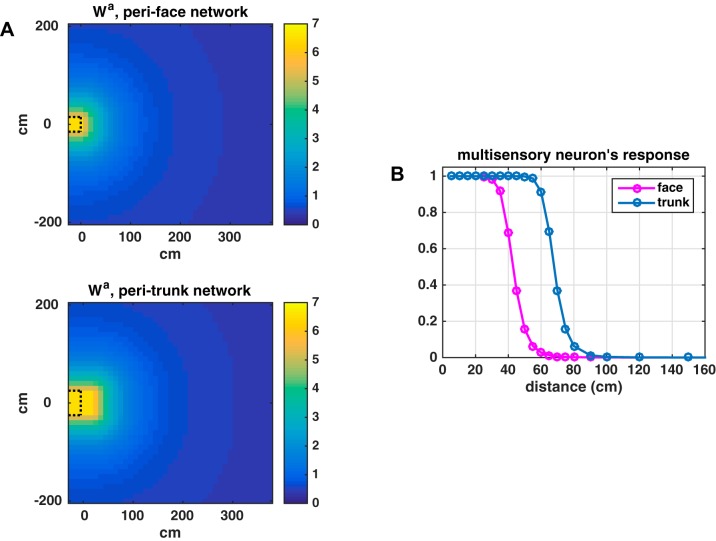
*A*: pattern of the feedforward synapses (*W^a^*) from the auditory neurons to the multisensory one in the peri-face space network and in the peri-trunk space network. The region limited by the dashed black line represents the body part extension in the frontal space (assumed equal to 20 cm). The frontal surface of the body part is at coordinate *x* = 0 cm. *B*: response of the multisensory neuron in the peri-trunk network (blue line) and in the peri-face network (magenta line) to a static sound placed at different distances from the body part (from 5 to 150 cm). The auditory stimulus lasted 100 ms with strength *S^a^* = 4.5. The figure depicts the maximum value reached by the multisensory neuron’s response to each static sound.

Figure 6*A* shows the obtained pattern of the auditory feedforward synapses for the trunk and the face (the same patterns hold for the feedback ones, rescaled so that the maximum value is $B_{0}^{a}$). Figure 6*B* displays the effect of the different synaptic patterns in the two networks, assessed by computing the peak activity reached by the multisensory neuron in response to a static sound (i.e., at a fixed position in space) lasting 100 ms. By using this stimulation, we excluded the effects of the continuity of sound in space, sound velocity (the stimulus being static as opposed to continuously moving in space), and neural adaptation (the stimulus being quick and not allowing for adaptation) and evaluated only the effect of the synaptic pattern. The peri-trunk space multisensory neuron responds to sounds at farther distances than the peri-face space multisensory neuron. It is important to specify that all other network components and mechanisms are equal in the two networks (the number of neurons, the lateral synapses, the tactile feedforward and feedback synapses, the input-output neuron relationship, and the adaptation mechanism).

### Neural Network Simulation and Performances

#### Simulation of the experimental tasks.

The networks were used to simulate the same experimental tasks performed in the behavioral section of the present study. This approach allows for evaluating whether the networks perform comparably to behavioral results, and, if so, to interpret behavioral observations in light of mechanistic network properties. The network is intended to represent a single hypothetical subject’s brain. The same set of simulations was replicated with the peri-face space and peri-trunk space network. Each set of simulations included unisensory tactile trials and multisensory audio-tactile trials. The stimuli resemble those used in vivo. The tactile stimulation was always an impulse stimulus lasting 100 ms. The auditory stimulus consisted in a looming sound starting at 200 cm distance from the skin surface and was simulated at four different velocities (25, 50, 75, 100 cm/s), rather than only the two utilized in the behavioral tasks (25 and 75 cm/s), to perform a broader analysis. In the audio-tactile trials, the tactile stimulus was applied at a given interval from the sound onset, depending on the sound velocity, so to replicate the seven sound-touch distances as in the behavioral experiments: 25, 50, 75, 100, 125, 150, 175 cm. Since behavioral data are expressed in terms of tactile RTs, a measure of the tactile reaction time must be decoded from the network. Network tactile RT was computed as the time necessary for the overall tactile activity (the sum of all tactile neurons’ activity) to reach a given threshold *L_th_* = 4 starting from the tactile stimulus onset. Since neuron activity ranges between 0 and 1, this means that an ensemble of a few tactile neurons needs to be active for the stimulus to be detected. In the multisensory condition, the activation in the tactile area can be speeded up compared with the unisensory condition, and network RT decreased, when the sound is able to trigger the multisensory neuron. To introduce variability in the network, in each trial the strengths of the stimuli (*S^t^*, *S^a^*) were randomly drawn from uniform distributions (mimicking sensory noise). In each condition (unisensory, and multisensory at each of the seven sound-touch distances and at each of the four velocities), 10 trials were simulated, both for the peri-face space and peri-trunk space network. Then, similarly to behavioral RTs, the network RTs in multisensory condition at the seven distances (i.e., 70 values of simulated RTs, 10 per distance), were fitted with a sigmoidal function (*Eq. 1*), separately for each velocity and body part. The parameters *y*_min_ and *y*_max_ were fixed a priori (the slowest and fastest median RT across the seven distances) and parameters *x_c_* (i.e., central point) and *b* (i.e., slope) were free to vary. In each condition (body part: face/trunk, velocity: 25, 50, 75, 100 cm/s), the fitting procedure provided the estimated value for the parameters *x_c_* and *b*, together with the 95% parameter confidence interval. The values of the parameter *x_c_* obtained from the network RTs were compared with those obtained behaviorally at 25 and 75 cm/s. Moreover, the in silico analyses at the four rather than two velocities were valued to predict how the behavioral proxy (*x_c_*) of PPS size is modulated by further stimulus velocity (e.g., in a gradual or steeper fashion). As for behavioral data, the fastest RT obtained in unisensory condition was used to correct the multisensory RTs, to quantify multisensory facilitation in tactile reaction time.

In a first stage, the above-described simulations were performed with parameters at their default values (Table 1). Then, identical simulations were ran while holding both *G^m^* and *G* equal to zero (“null model”) to ascertain whether the adaptation mechanism is the key contributor to the dynamic resizing of PPS as a function of sound velocity. Subsequently, to assess robustness of the model outcome, sensitivity analyses were performed by replicating the simulations described above while altering the values of neural adaptation parameters (*G*, *G^m^*, *T*, τ). Further sensitivity analyses on additional model parameters (e.g., those governing the auditory stimuli—Intensity or *S_a_*, and spatial precision or $\sigma_{I}^{a}$—and those governing synaptic connections among the auditory and multisensory areas—parameters *k*_1_, *k*_2_, and *X_c_*) were executed as an indication that it is the neural architecture and the presence of a neural adaptation mechanism that engenders the resizing of PPS as a function of sound velocity, and not the particular parameters chosen. Lastly, simulations with static sounds were executed to ascertain whether a PPS effect can be engendered with sounds that do not dynamically move in space.

#### Sensitivity analyses.

Regarding sensitivity of the network to the stimuli chosen, a single trial was simulated with the peri-face and peri-trunk networks while sounds approached at 25, 50, 75, and 100 cm/s and tactile stimulation was given when the sound was at a distance of 25, 50, 75, 100, 125, 150, and 175 cm. All parameters were kept at their default values (mostly inherited from previous work—Magosso et al. 2010b; Serino et al. 2015b—and exposed in Table 1), with exception of *S^a^* and $\sigma_{I}^{a}$, which were parametrically adjusted. Similarly, for contrast purposes, the peri-face network was ran while nullifying the adaptation mechanism (*G^m^* and *G* were zeroed), while the peri-trunk network was run with the default adaptation mechanism in place. Similarly, regarding synapse parameters, sensitivity analyses were performed for three distinct variables: *k*_1_ and *k*_2_, which dictate the shape (i.e., the slope) of the exponential function ruling the pattern of connections between unisensory auditory neurons and the multisensory neuron (see Table 1 and *Eqs. 5*–7), and $X_{C}^{F}$ or $X_{C}^{T}$, for the peri-face and peri-trunk networks, respectively, which defines the extension in the frontal space (i.e., along *x*) of the region where auditory synapses keep the maximal value (i.e., beyond the upper extreme of this region, synapses start to decrease; e.g., see Fig. 6*A*, and *Eqs. 5*–7). This last parameter effectively is the one differentiating the peri-face and peri-trunk networks.

#### Simulations with static sounds.

Additional simulations explored the effects of static (i.e., not moving in space) sounds of different intensities. Indeed, the notion that PPS size may putatively be modulated by the level of activation of neurons in the auditory area (influencing and via influences from the multisensory area) equally applies to the case of stimuli of null velocity (static) and of different intensities, which engender different levels of auditory activation because of their different strength. Accordingly, audio-tactile trials with static sounds applied at same seven distances utilized in the rest of simulations and having a variable intensity were performed. Specifically, two sets of new audio-tactile simulations have been performed. For briefness, only the peri-trunk network was tested. In the first set of simulations, an auditory stimulus of a fixed intensity was applied in a stable position at a given distance from the body part, and a tactile stimulus was delivered during sound presentation. The auditory stimulus was 200 ms long and the tactile stimulus was 100 ms long; application of touch was delayed by 100 ms from sound onset. Each of the seven sound-touch distances (25, 50, 75, 100, 125, 150, 175 cm) were tested at four different intensities of the auditory stimulus (*S^a^* = 3, 5, 7, 9; here no noise was applied on auditory stimulus intensity as we precisely wanted to assess its effect). Intensity of tactile stimulus was affected by uniform noise [*S^t^* drawn from a uniform distribution in the range (3.3–3.7)]. Ten trials were simulated for each sound intensity and distance, and tactile RT was computed for each trial. By adopting the same procedure used to estimate PPS size in case of moving stimuli, the tactile RT obtained in each multisensory condition was corrected for the fastest unimodal tactile RT, to get tactile facilitation; values of tactile facilitation were then fitted by a sigmoidal function, separately for each sound intensity. In a second set of simulations with static sounds the intensity of the static sound was modulated within each presentation. More precisely, the auditory stimulus was applied in a stable position at a given distance from the body part, and its intensity was progressively increased from 0 to 10 over 2 s. During each auditory stimulation, the tactile stimulus [lasting 100 ms and with intensity drawn from a uniform distribution in the range (3.3–3.7)] was delivered at a different time from sound onset, so that it occurs when the auditory stimulus had reached either intensity 3, 5, 7, or 9. All the seven sound-body distances (25, 50, 75, 100, 125, 150, 175 cm) were tested (here, 5 trials were replicated for each position and each time of tactile presentation).

#### Simulation of neurophysiological data in literature.

A second set of simulations were performed to relate our behavioral proxy of PPS size (the central value of the sigmoidal function fitting tactile RT in multisensory condition) with a neural measure of the PPS size, that is, the size of the multisensory neuron’s auditory RF estimated from the multisensory neuron’s response to approaching sounds. In particular, this analysis was directly inspired by Fogassi and colleague’s seminal paper (Fogassi et al. 1996) showing that the visual RF of multisensory visual-tactile neurons increased in depth as the velocity of the incoming visual stimulus increased from 20 to 80 cm/s. Here, we asked whether the network reproduces size modulation of the multisensory neuron’s auditory RF as a function of sound velocity, similarly to that reported in Fogassi’s paper. That is, making use a latent variable of the network (i.e., the activity of the multisensory neuron), we replicate the exact electrophysiological analysis as in Fogassi et al. (1996) (except that we used auditory probing stimuli, instead of visual ones). It must be noted, thus, that this last analysis does not rely on an output function—from neural response to behavioral outcome—but directly indexes RF size based on (simulated) neural activity, as done in Fogassi et al. (1996).

To this aim, we performed simulations consisting of a sound alone moving at different velocities and, based on the response of the multisensory neuron, the size of its auditory RF was estimated at each sound velocity, by adopting the same computation as in Fogassi et al. (1996). In particular, at each sound velocity, we computed the sound distance at which the multisensory neuron started to respond; namely, the onset of multisensory neuron response being considered as the bending point (i.e., where the slope changes) of the cumulative sum of the neuron’s activity. The so-computed sound distance was taken as to index the size of the multisensory neuron’s auditory RF. To perform this computation, we calculated the cumulative sum (CS) of the multisensory neuron’s activity, normalized by the overall number of simulation steps. Then, the cumulative sum of the multisensory neuron activity as a function of sound distance (say CS(*D*), with *D* = 200 cm → 0 cm) was fit with a two-segment piecewise linear function, as in Fogassi et al. 1996. One segment of the fitting function connected the point {*D*_o_ = 200 cm, CS(*D*_o_)} with the point {D*, CS(D*)}, while the other segment connected the point {D*, CS(D*)} with the point {*D*_end_ = 0 cm, CS(D_end_)}. Parameter D*, representing the bending point, was free to vary between 200 and 0 cm. The value of D* performing the best fitting (minimum sum of squared errors), signaled the change in the slope of CS(*D*) and was taken as the size of the multisensory neuron’s auditory RF [mimicking Fogassi et al. (1996)'s analysis].

Size estimation of the multisensory neuron’s auditory RF was performed at the same four velocities used in RT simulations (25, 50, 75, 100 cm/s), as well as at lower (12.5 cm/s) and higher (125, 150, 200 cm/s) velocities, to infer a more complete description of the relationship between RF size and stimulus velocity.

## RESULTS

### Behavioral Results

#### Accuracy.

**Fig. 7.**
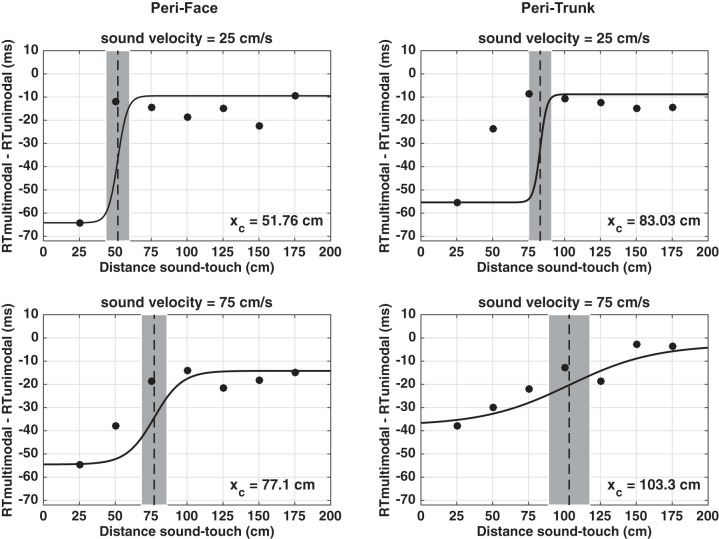
Behavioral data depicting peri-face and peri-trunk space as a function of sound velocity (25 vs. 75 cm/s). Audio-tactile facilitation (*y*-axis, in ms), i.e., multisensory reaction time (RT)–unisensory RT (which by definition is equal to zero) is illustrated as a function of sound-touch distance (*x*-axis, in cm), i.e., distance of the sound from the body part. For graphical purposes, the median RTs for each body part and at each velocity were fit with a sigmoidal function (*Eq. 1*) whose central point was taken as the average of the central points estimated on individual subjects in the corresponding condition (thick dashed vertical line). In each panel, the shaded area surrounding the average of the estimated central points represents the 95% confidence intervals (CI).

Overall participants demonstrated to be very accurate at withholding response during unimodal auditory catch trials (peri-face, M = 99.02%, SE = 1.09%; peri-trunk, M = 99.08%, SE = 1.10%), and thus results were solely analyzed in terms of RTs (see Fig. 7).

#### Space-dependent multisensory effect.

With regard to RTs, the initial 2 (body part: Face vs. Trunk) × 2 (velocity: 25 cm/s vs. 75 cm/s) × 7 (Sound Distance: D1 through D7) mixed-model ANOVA showed a significant three-way interaction (*F*_6,174_ = 2.58, *P* = 0.017, partial η^2^ = 0.16, Greenhouse-corrected), and thus results were bifurcated by body part.

With regard to the RTs to touch on the face as a function of the distance of task-irrelevant sounds, a repeated-measures ANOVA demonstrated a main effect of distance (*F*_6,90_ = 41.56, *P* < 0.001, partial η^2^ = 0.73), and a distance × sound velocity interaction (*F*_6,90_ = 3.79, *P* = 0.002, partial η^2^ = 0.20), yet no main effect of sound velocity (*F*_1,15_ < 1, *P* = 0.99). The significant interaction is explained by the fact that in the case of slow sounds audio-tactile RTs were faster than unisensory tactile reaction times only at D1 (Wilcoxon signed-rank test against a hypothesized null distribution with median = 0, *Z* = 3.46, *P* < 0.001) but at none of the other distances (all *P* > 0.07). In the case of fast sounds, at D1 (Wilcoxon signed-rank test, *Z* = 3.36, *P* < 0.001), D2 (Wilcoxon signed-rank test, *Z* = 2.22, *P* = 0.026), and D3 (Wilcoxon signed-rank test, *Z* = 2.06, *P* = 0.039) audio-tactile RTs were faster than purely tactile RTs (statistical comparison is to a median = 0). At the remaining distances unisensory and multisensory RTs were equivalent. In addition, overall, RTs in multimodal experimental trials were faster than those in baseline unimodal trials [25 cm/s; median (M) = −19 ms, Wilcoxon signed-rank test against zero, *Z* = 2.12, *P* = 0.034; 75 cm/s; M = −20 ms, Wilcoxon signed-rank test against zero, *Z* = 2.14, *P* = 0.032]. Hence, audio-tactile RTs were both multisensory, in that there was a significant facilitation vis-à-vis unimodal RTs, and modulated as a function of the spatial disparity between the two. Thus, via our audio-tactile task we were able to capture the representation of peri-face space.

Similarly, in the case of sounds directed toward the trunk, repeated-measures ANOVA demonstrated a main effect of distance (*F*_6,84_ = 9.51, *P* < 0.001, partial η^2^ = 0.40), and a distance × sound velocity interaction (*F*_6,84_ = 1.99, *P* = 0.042, partial η^2^ = 0.13), yet no main effect of sound velocity (*F*_1,14_ < 1, *P* = 0.71). The significant interaction is explained by the fact that in the case of slow sounds when audio was presented at D1 (Wilcoxon signed-rank test against a hypothesized null distribution with median = 0, *Z* = 3.06, *P* = 0.002) and D2 (*Z* = 2.55, *P* = 0.011) reaction times to audio-tactile stimulation were significantly faster than to purely tactile stimulus. When audio was presented furthermore, there was no significant facilitation in RT as a consequence of presenting a multisensory (vs. unisensory) condition (all *P* > 0.23). In the case of fast sounds, D1 (*Z* = 3.23, *P* = 0.001), D2 (*Z* = 2.38, *P* = 0.017), and D3 (*Z* = 2.89, *P* = 0.004) were all faster than unimodal tactile stimuli (e.g., multisensory facilitation), while the remaining audio-tactile distances showed no multisensory facilitation. Furthermore, RTs to touch administered on the trunk were in general significantly faster when sounds were present (25 cm/s; M = −18.4 ms, *Z* = 2.21, *P* = 0.025; 75 cm/s; M = - 10.9 ms, *Z* = 1.98, *P* = 0.047) vs. absent.

#### Sigmoidal fittings.

The sigmoidal functions proved to adequately and equally describe the relation between RTs to touch and distance between auditory and tactile stimulation across conditions (peri-face; 25 cm/s, *R*^2^ = 0.54 ± 0.06, 75 cm/s, *R*^2^ = 0.50 ± 0.07; peri-trunk; 25 cm/s, *R*^2^ = 0.46 ± 0.06, 75 cm/s, *R*^2^ = 0.44 ± 0.06; mixed model ANOVA, all *P* > 0.12).

We then analyzed the location of the central point of the sigmoidal function as an index of the location of the PPS boundary in each condition (see Serino et al. 2015a). The mixed model ANOVA revealed main effects of body part mapped (*F*_1,29_ = 18.6, *P* < 0.001, partial η^2^ = 0.39), and sound velocity (*F*_1,29_ = 13.70, *P* = 0.001, partial η^2^ = 0.32). No interaction between these variables was observed (*P* = 0.84). As depicted in Fig. 7, the main effect of sound velocity was present both for the peri-face (*left*; 25 cm/s, M = 51.76 cm; 75 cm/s, M = 77.1 cm; *Z* = 2.32, *P* = 0.02) and the peri-trunk (*right*; 25 cm/s, M = 83.03 cm; 75 cm/s, M = 103.3 cm; *Z* = 2.21, *P* = 0.027), showing that for both representations, the PPS boundary was located further in space when probed with faster as compared with slower sounds. In addition, both for the 25 cm/s condition (*top*; Mann-Whitney *U*-test between groups, *U* = 196, *P* = 0.002) and 75 cm/s condition (*bottom*; Mann-Whitney *U*-test between groups, *U* = 199, *P* = 0.001), the representation of peri-trunk space was larger than that for peri-face space (see Serino et al. 2015b for a similar finding).

In terms of the slope describing the transition between peri- and extrapersonal space, the mixed model ANOVA revealed a main effect of body part (*F*_1,29_ = 5.30, *P* = 0.029, partial η^2^ = 0.15), yet no main effect of sound velocity (*F*_1,29_ = 0.38, *P* = 0.53), nor an interaction between body part mapping and sound velocity (*F*_1,29_ = 0.05, *P* = 0.81). The main effect of body part was explained by a shallower boundary for the peri-trunk space (M = 74.03) than for the peri-face space (M = 41.76; Mann-Whitney *U*-test between groups, *U* = 176, *P* = 0.027).

### Neural Network Results: Simulation of the Experimental Tasks

**Fig. 8.**
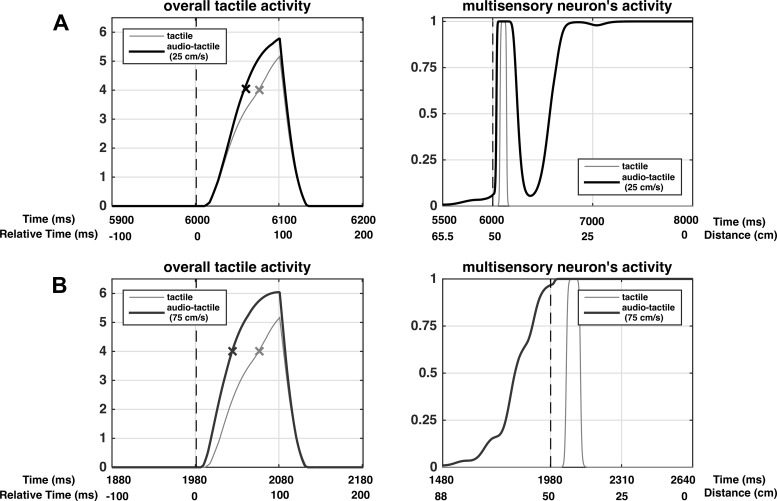
Exemplary patterns of activations in the peri-face space network in case of a unisensory tactile trial (stimulus duration 100 ms, strength *S^t^* = 3.5, gray lines) and multisensory audio-tactile trial with a looming sound at 25 (*A*) and 75 cm/s (*B*). In the audio-tactile trial, the moving sound has strength *S^a^* = 7; the tactile stimulus is the same as in the unisensory trial, applied when the sound is at distance *D* = 50 cm (*t* = 6,000 ms in *A* and *t* = 1,980 ms in *B*). *Left*: the overall tactile activity (the sum of all tactile neurons’ activity), zoomed between −100 ms and +200 ms around the tactile stimulus onset (vertical dashed black line). The × on each curve marks the instant at which the overall tactile activity overcomes the detection threshold (*L_th_* = 4). *Right*: multisensory neuron’s response as a function of both time and distance of the sound from the body part; the tactile stimulus onset (vertical dashed black line) is displayed too, for convenience.

Figure 8 shows exemplary peri-face space network responses to a unisensory tactile trial and multisensory audio-tactile trial. In the audio-tactile trial, the looming sound moved at 25 cm/s (Fig. 8*A*) and 75 cm/ s (Fig. 8*B*), and the tactile stimulus was applied when the sound was at distance *D* = 50 cm (*t* = 6,000 ms and *t* = 1,980 ms, respectively). For the sake of comparison, the tactile stimulus in unisensory condition was applied at the same time instants. Both the slower and faster sounds contribute to reduce tactile RT, i.e., the overall tactile activity reaches the detection threshold earlier compared with unimodal condition. However, the facilitation effect is larger in case of the faster than slower sound. Indeed, as the slow sound approaches the distance *D* = 50 cm, it provides an auditory input to the multisensory neuron insufficient to trigger its activation (the multisensory neuron is working in the lower portion of its sigmoidal activation function), and the tactile area is not receiving any preparatory feedback from the multisensory neuron. Only when the tactile stimulus is applied and the multisensory neuron is quickly activated (via the sum of the tactile and auditory input), the tactile neurons receive a feedback input that speeds up their activation. Conversely, when the sound at 75 cm/s approaches the distance *D* = 50 cm, the multisensory neuron is already led to its maximum activity, and sends back a “preparatory” feedback input to the tactile neurons, well before the application of the tactile stimulus. This feedback input moves the working point of the tactile neurons along the low-saturation region closer to the high-gain portion of the activation function; thus, when the tactile stimulus is applied, tactile activation is both anticipated and speeded up. This different behavior at the two sound velocities arises from the different level of activity in the auditory area (see Fig. 5).

**Fig. 9.**
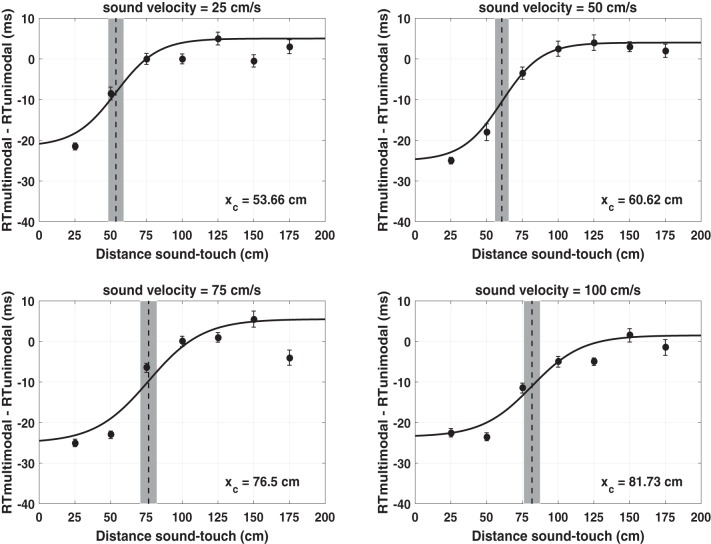
Simulated tactile reaction times (RTs) with the peri-face space network as a function of the looming sound velocity. In each panel, values of audio-tactile facilitation, i.e., tactile RTs obtained in multisensory condition corrected by the fastest unisensory tactile RT, are reported as a function of sound-touch distance. Ten audio-tactile trials were replicated with the network at each sound-touch distance and at each velocity: in each trial, the strength of the tactile stimulus and the strength of the auditory stimulus were drawn from a uniform distribution in the ranges 3.3–3.7 and 6–8, respectively. The point and error bars at each sound-touch distance represent mean RT ± 1 SE. For each sound velocity, the overall RTs (70 points) were fitted with a sigmoidal function (*Eq. 1*). The dashed vertical line marked the estimated value of the central point, and the surrounding shaded area represents the 95% confidence interval of the estimated parameter. *X_C_*, central point of the sigmoidal function.

The overall results of RT simulations performed with the peri-face space network are reported in Fig. 9. At the shortest sound-touch distance (*D* = 25 cm), multisensory tactile facilitation is as high as 20–25 ms, and is independent of sound velocity. At large sound-touch distances (*D* ≥ 100 cm), multisensory facilitation is almost null at all tested sound velocities. Sound velocity mainly modulates tactile RTs between 50 and 75 cm sound-touch distances. Indeed, tactile RT gradually decreases by ~10–12 ms at both distances, as sound velocity increases from 25 cm/s (Fig. 9, black) to 100 cm/s (Fig. 9, blue). According to these results, at short distances (*D* ≤ 25 cm), the feedforward auditory synapses are high enough so that even lower levels of auditory activity induced by slow sounds bring the multisensory neuron to its maximum activity, thus providing maximum RT facilitation. At far distances (*D* ≥ 100 cm), the feedforward auditory synapses are so low that even high auditory activities induced by faster sounds are unable to elicit multisensory neuron response, thus providing no facilitation. At intermediate distances, where the transition between high and low synaptic values occurs (Fig. 6*A*, *top*), the level of activity in the auditory area is critical in modulating multisensory neuron activation and RT facilitation. By looking at the sigmoidal function fitting the simulated RTs (25 cm/s: *R*^2^ = 0.74; 50 cm/s: *R*^2^ = 0.82; 75 cm/s: *R*^2^ = 0.78; 100 cm/s: *R*^2^ = 0.83), two main considerations can be drawn: *1*) The modulation of the RTs by the sound’s velocity at *D* = 50 cm and 75 cm results in a progressive increase of the central point of the sigmoidal function from ~54 cm to ~82 cm as the velocity increases from 25 to 100 cm/s. *2*) The values of the central point estimated from the network RTs in the audio-tactile trials at 25 and 75 cm/s sound velocity are in line with those obtained from the experimental RTs (see Fig. 11 for a qualitative comparison).

**Fig. 10.**
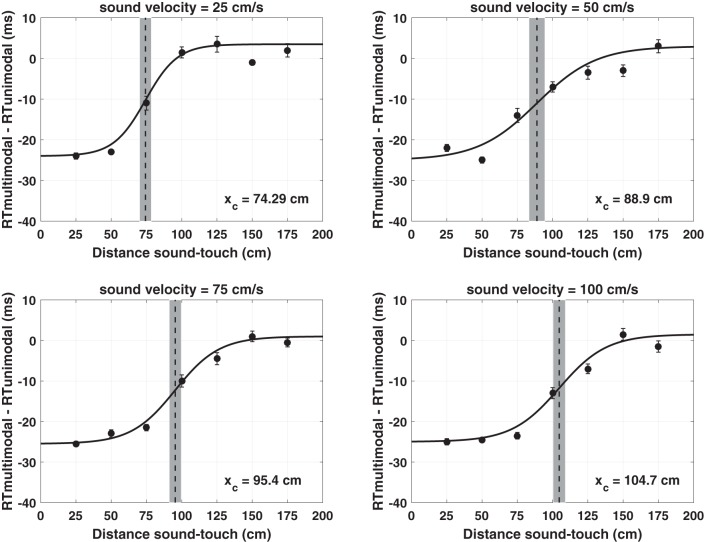
Simulated tactile reaction times (RTs) with the peri-trunk space network as a function of the looming sound velocity. The displayed results were obtained as in the peri-face space network, and the meaning of the symbols is the same as in Fig. 9. *X_C_*, central point of the sigmoidal function.

**Fig. 11.**
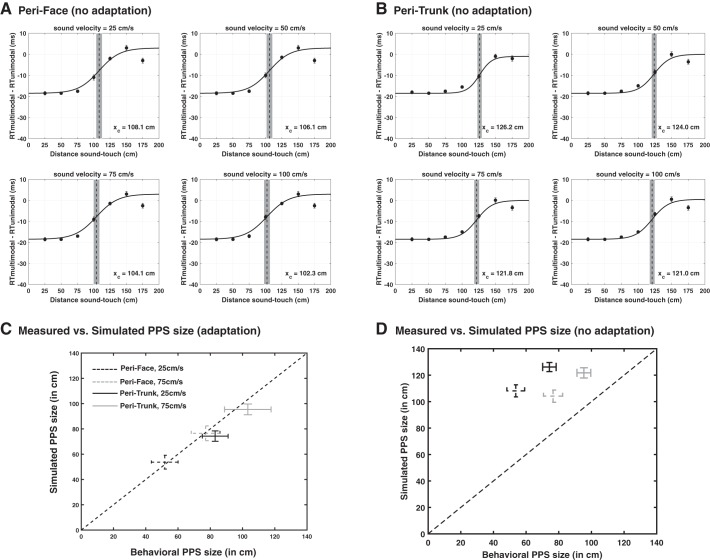
*A*: simulated tactile reaction times (RTs) with the peri-face network as a function of the looming sound velocity when the neural adaptation mechanism is nullified. Each subpanel has been obtained adopting exactly the same procedure as in Fig. 9 (the only difference is that here the adaptation mechanism is not operating). The meaning of the symbols and lines is the same as in Fig. 9. *B:* same as *A* with exception that peri-trunk RTs were simulated (again, with the adaptation mechanism nullified). *C*: comparison between estimates of central points for the different body parts and velocities produced behaviorally and through neural network simulation when the adaptation mechanism was operative. *D*: comparison between estimates of central points for the different body parts and velocities produced behaviorally and through neural network simulation with no adaptation mechanism. *X_C_*, central point of the sigmoidal function; PPS, peripersonal space.

Figure 10 displays the results of RT simulations performed with the peri-trunk space network. Here, the sound velocity mainly modulates tactile RTs between 75- and 100-cm sound-touch distances. RT Facilitation by ~20–23 ms at lower distances (*D* ≤ 50 cm) and absence of facilitation at higher distances (*D* ≥ 125 cm) are preserved at each sound velocity. This agrees with the different pattern of the auditory feedforward synapses in the peri-trunk space network, where the transition from high to low synaptic values occurs at farther distances than in the peri-face space network (Fig. 6*A*). The results of the fitting procedure (25 cm/s: *R*^2^ = 0.86; 50 cm/s: *R*^2^ = 0.83; 75 cm/s: *R*^2^ = 0.88; 100 cm/s: *R*^2^ = 0.88), indicate the following: *1*) The central point of the sigmoidal function progressively increases from ~74 to ~105 cm as velocity increases. *2*) At each sound velocity, the central point of the sigmoidal function assumes a higher value in the case of the peri-trunk space than peri-face space network. *3*) The values of the central point estimated from the simulated RTs in the audio-tactile trials at 25 and 75 cm/s sound velocity are in line with those estimated from the experimental RTs (see Fig. 11 for a qualitative comparison).

### Neural Network Results: Simulation of the Experimental Tasks Without Adaptation and Sensitivity Analyses

To ascertain that the modulation in PPS size replicated via neural network simulation was due to the neural adaptation implemented, we compared the above-mentioned model, to a “null” model, with no adaptation mechanism—formally, parameters *G^m^* and *G* in *Eq. 4*, setting the strength of adaptation, were set to zero. The simulation of this null model was effectuated in an identical manner to that of the full model described throughout the paper. As depicted in Fig. 11, *A* and *B* for the peri-face and peri-trunk respectively, when the adaptation mechanism was nullified, sound velocity did not appreciably modulate the size of PPS. In fact, for the face, when sound velocity was set to 25 cm/s, peri-face size was estimated at 108.1 cm, a value that decreased monotonically until 102.03 cm when sounds approached at 100 cm/s. This last value i.e., central point of peri-face function when sounds were equal to 100 cm/s was within the lower bound of the 95% confidence interval (CI) for the simulations with 50 cm/s (101.52 cm) and 75 cm/s (110.63 cm) sounds (see Fig. 11*A*). Similarly, the peri-trunk space was estimated at 126.2 cm when sounds approached at 25 cm/s and decreased by ~5 cm when sounds quadrupled in speed. Again, when the adaptation mechanism was nullified, sound velocity did not significantly modulate the size of the peri-trunk space (estimate for peri-trunk size at 100 cm/s within the 95% CI for 50 and 75 cm/s; see Fig. 11*B*). To qualitatively contrast the null model (e.g., no adaptation) with the adaptation model, we adopted a bootstrapping approach wherein bivariate distributions were centered in a two-dimensional space at the measured (*x*-axis) and simulated (*y*-axis) peri-face and peri-trunk space sizes for 25 and 75 cm/s. The dispersion of these Gaussians were set equal to the standard deviations of the measured and simulated PPS sizes, and we randomly drew 1,000 observations from these distributions. In Fig. 11*C* (adaptation) and D (no adaptation) we plot in a two-dimensional space the mean and 95% CI the simulated PPS size (*y*-axis) as a function of the behaviorally measured PPS size (*x*-axis). The point here is that the simulated data accounting for neural adaptation is closer to the identity line than the simulated data without adaptation (while all other parameters are kept constant). Of not, the statistical comparison between simulated data and in vivo data is unwarranted, as in principle this fit can be arbitrarily good by manipulating parameter values. Therefore, rather than statistically comparing model outcomes to behavioral outcomes, sensitivity analyses were performed to assess robustness of model’s results against modifications in its parameter values.

**Fig. 12.**
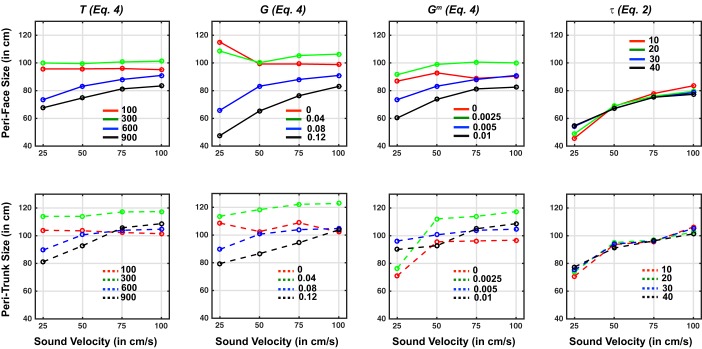
Analysis of the sensitivity of the simulated results to the particular values adopted by parameters governing neural adaptation. *Top*: sensitivity analysis in the peri-face network. *Bottom*: sensitivity analysis in the peri-trunk network. Within each subpanel, *x*-axis represents the sound velocity simulated (25, 50, 75, or 100 cm/s) and the *y*-axis represents the size of the peripersonal space (PPS) engendered by the simulation. First column shows size of PPS as a function of *T* between 100 ms and 900 ms. This parameter dictates the temporal interval over which neural adaptation may occur (*Eqs. 4*). Second and third column, respectively, depict the size of PPS as a function of the positive gain parameter that establishes the amount of adaptation, respectively, in auditory (2nd column) and multisensory (3rd column) neurons. Lastly, the fourth column illustrates the size of PPS as a function of sound velocity and the particular value of τ and τ*^m^* (these two always being kept equal). These parameters dictate the temporal constant with which input to neurons affect their output, respectively, in unisensory and multisensory areas. See Table 1 for definitions and values of *T*, *G*, *G^m^*, τ, and τ*^m^*.

Importantly, as shown by a sensitivity analysis (Fig. 12) on the neural adaptation parameters, the presence of the adaptation mechanism was necessary to account for velocity modulation of PPS size; however, the particular values adopted by the mechanism were not. Keeping the rest of parameters at their default values, a single trial was simulated for the peri-face and peri-trunk space while parametrically altering values for the parameters governing neural adaptation (*T*, *G^m^*, *G*, and τ and τ*^m^*; see Table 1 for their default values). Approaching sounds were simulated at 25, 50, 75, and 100 cm/s, and reaction times were extracted at 25, 50, 75, 100, 125, 150, and 175 cm. These reaction times were then fit to a sigmoidal function and the central point of this sigmoid was taken as the spatial extension of the PPS. As suggested in Fig. 12 (*top*: peri-face; *bottom*; peri-trunk), while a minimal value is necessary to allow the neural adaptation mechanism to come into play, the pattern of results (larger PPS with faster sounds) remains constant thereafter irrespectively of the particular values chosen.

**Fig. 13.**
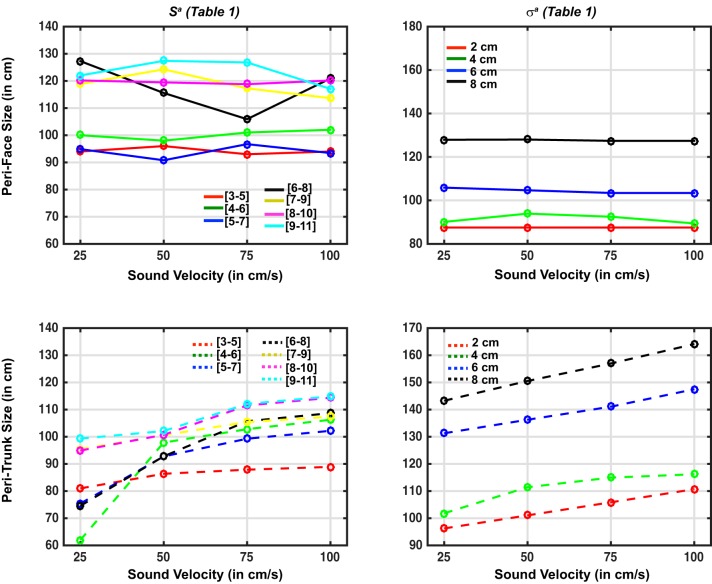
Analysis of the sensitivity of the simulated results to the particular values adopted by parameters governing auditory stimuli (parameter *S^a^*, and parameter $\sigma_{I}^{a}$; see Table 1 for their basal value). *Top*: sensitivity analysis in the peri-face network (in which the neural adaptation mechanism was nullified; negative control). *Bottom*: sensitivity analysis in the peri-trunk network (with the neural adaptation mechanism in place; positive control). Within each subpanel, *x*-axis represents the sound velocity simulated (25, 50, 75, or 100 cm/s) and the *y*-axis represents the size of the peripersonal space (PPS) engendered by the simulation. First column shows size of PPS as a function of *S^a^*, the intensity of auditory stimulation, between 3 and 5 (uniform distribution) and 9 and 11 (uniform distribution). The second column depicts the size of PPS as a function of the standard deviation of the auditory stimuli—its spatial localizability—from 2 (red) to 8 cm (black).

**Fig. 14.**
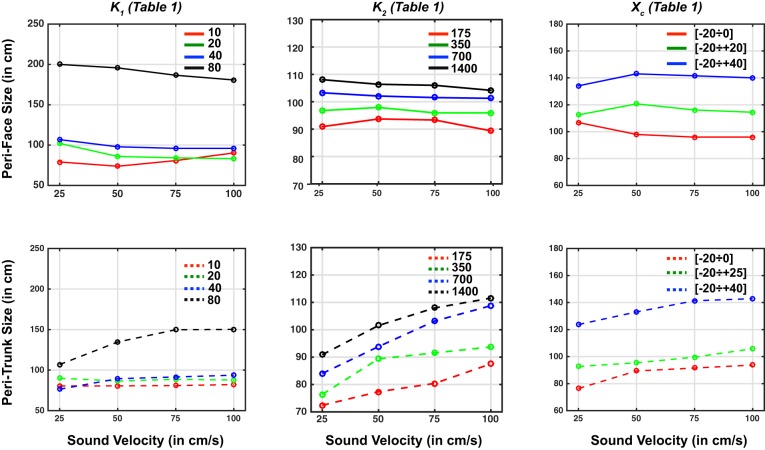
Analysis of the sensitivity of the simulated results to the particular values adopted by parameters governing synaptic connections (parameters *k*_1_, *k*_2_, $X_{C}^{F}$, $X_{C}^{T}$; see Table 1 for their basal value). *Top*: sensitivity analysis in the peri-face network (no neural adaptation; negative control). *Bottom*: sensitivity analysis in the peri-trunk network (with neural adaptation; positive control). Within each subpanel, *x*-axis represents the sound velocity simulated (25, 50, 75, or 100 cm/s) and the *y*-axis represents the size of the peripersonal space (PPS) engendered by the simulation. The first and second columns show sensitivity of PPS size as a function of *k*_1_ and *k*_2_, which dictate the slope of the exponential function ruling the connections between the auditory neurons and the multisensory neuron. The third column depicts the size of PPS as a function of sound velocity and the particular value of $X_{C}^{F}$ for the peri-face (negative control) and $X_{C}^{T}$ for the peri-trunk network (positive control). This parameter defines the extension in the frontal space (i.e., along *x*) of the region where auditory synapses keep the maximal value.

Figures 13 and 14 show the results of the sensitivity analysis on auditory stimuli parameters and synapse-related parameters. As depicted in Fig. 13 (*top left* and *bottom*) the intensity of the auditory stimulation (*S^a^*) tends to affect the overall prediction of the PPS size, the latter enlarging as stimulus intensity increases, both for the trunk and for the face. Most importantly, although the measure is variable (due to the fact that a single trial was simulated and uniform noise was introduced (in the case of 3–5, red line, noise and signal are of similar magnitude), the strength of auditory stimuli did not seemingly interact with the sound velocity effect, which is present when the neural mechanism is present (peri-trunk network) and absent when the neural adaptation mechanism is nullified (peri-face network). Interestingly, on the other hand when the standard deviation of the auditory stimuli—analogous to the reliability of sound localization—was increased from 2 to 8 cm (default value being 6 cm; see Table 1)—the size of both the peri-face (*top right*) and peri-trunk (*bottom right*) enlarged. This observation is well in line with the characterization of PPS as a predictive mechanism (Kandula et al. 2017; Noel et al. 2017; Roncone et al. 2016); however, it remains but a speculation here, due to the fact that no behavioral data is put forward. This prediction could be tested in future behavioral tasks. Regarding synaptic parameters, as depicted in Fig. 14 manipulation of these parameters predictably changed the overall size of the PPS representation, but did not necessarily obviate the effect of sound velocity (see the peri-trunk space, Fig. 14, *bottom*). Similarly, the negative control in the peri-face network demonstrated that when the neural adaptation mechanism was not present, regardless of the values of the parameters governing synaptic behavior, no velocity effect was present. These simulations, as for the stimuli parameters, are noisy, likely due to the fact that a single trial was simulated due to computational cost. Taken together, these simulations confirm that when the adaptation mechanism is in operation, velocity-dependent PPS resizing is overall maintained despite parameter fluctuations. Indeed, although fluctuations in these parameters change the precise PPS size prediction, they do not alter the impact of neural adaptation on PPS and in engendering a PPS velocity effect (compare Figs. 13 and 14, *bottom* vs. Figs. 13 and 14, *top*). Furthermore, these analyses provide additional interesting predictions. Of particular interest are the simulations in Fig. 13, *bottom*, suggesting that, while an increased sound intensity scales PPS size positively (Fig. 13, *bottom left*), an increased precision in sound location (decrease in standard deviation) shrinks the size of PPS (Fig. 13, *bottom* *right*). It would be interesting to test these latter predictions in future experimental work.

### Neural Network Corollary Results: Effects of Static Sounds of Variable Intensity

**Fig. 15.**
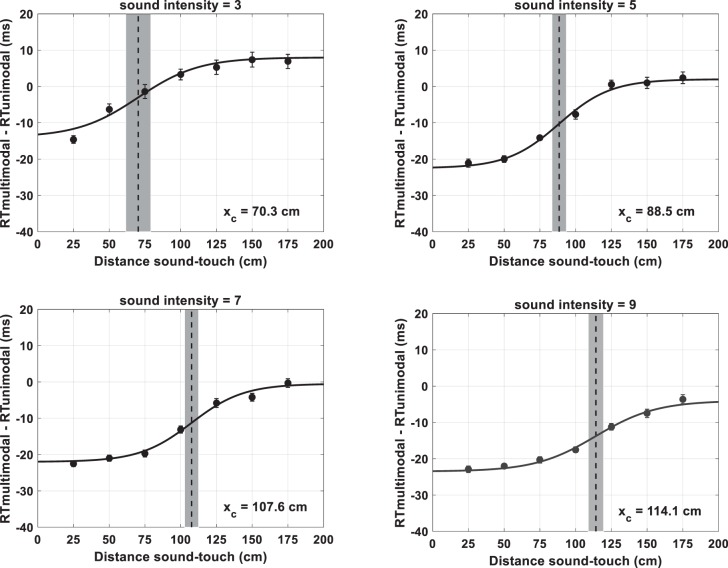
Results of the first set of simulations performed with the peri-trunk network using static sounds. Each panel shows the simulated values of tactile facilitation at the seven sound-touch distances for each of the four tested intensities of the sound. In each trial, the sound (200-ms duration) was stable at a given distance and at a given intensity; during sound presentation, the tactile stimulus (100-ms duration) was delivered with a stimulus onset asynchrony of 100 ms. Since tactile stimulation was affected by noise, 10 trials were replicated at each sound-touch distance and at each sound-intensity; each point represents mean ± SE. For each sound intensity, the overall facilitation values (70 values) were fitted with a sigmoidal function (*Eqs. 1* in main text). The dashed vertical line marked the estimated value of the central point (taken as a proxy of PPS size), and the surrounding shaded area represents the 95% confidence interval of the estimated parameter.

Complementary to the main aim of the present paper (speed-dependent modulation of PPS), we used our modeling tool to predict whether PPS resizing may occur even in case of static sounds when their intensity (an attribute that modifies the level of activation in the auditory area, parameter *S^a^*) was changed. As illustrated by Fig. 15, at each sound intensity, tactile facilitation is modulated by the sound-touch distance, i.e., a PPS effect is generated. This result is important since both at the neurophysiological level (e.g. see Graziano et al. 1997, Fig. 4*D* in that paper) and at psychophysical level (e.g., see Farné and Làdavas 2002; Serino et al. 2007 with reference to auditory stimulation), peripersonal space representation has been found to emerge even with exteroceptive static stimuli, and it is in line with our previous modeling works simulating the effect of static stimulations (Magosso et al. 2010a, 2010b). Second, the PPS size is modulated by the intensity of the static auditory stimulus: the size of the PPS expands as the intensity of the static stimulus increases, in a fashion similar to that observed for increasing speed of dynamic sounds.

**Fig. 16.**
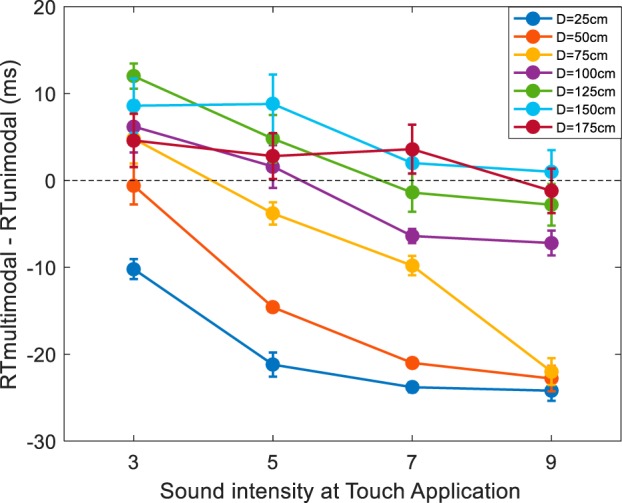
Results of the second set of simulations performed with the peri-trunk network using static sounds. In each trial, the sound was kept stable at a given distance while its intensity was linearly increased from 0 to 10 over 2 s and the touch was applied at a proper time from sound onset so to occur when the sound had reached one of the four intensities (3, 5, 7, 9). For each sound-body distance, tactile facilitation (mean ± SE over 5 trials) is depicted as a function of the sound intensity at touch application. RT, reaction time.

The obtained values of tactile facilitation (mean ± SE) are displayed in Fig. 16 as a function of sound-touch distance and as a function of sound intensity at the time of tactile stimulation. At all sound-touch distances but the largest one (*D* = 175 cm, at which all intensities provide similar values), the progressive increase in sound intensity progressively enhances tactile facilitation, as the PPS size enlarges simultaneously with sound intensity. This is especially evident for distances in the range 25–100 cm. Of course (and in line with results in Fig. 15), sounds at farther distances provides overall smaller facilitation than closer sounds. This set of simulations further supports that, according to the model, static sounds are able to elicit PPS effects and modulation of their attributes (e.g., intensity) can dynamically remap PPS. Of note, however, even at the strongest sound intensities simulated, far sounds did not result in a multisensory facilitation that is comparable to near sounds. In fact, sounds presented at the closest distance (*D* = 25 cm) were further facilitative of tactile processing than sounds presented at far distances (*D* = 100 cm and above) and at the highest intensity simulated (see Fig. 16). Nonetheless, these results highlight the methodological importance of using stimuli that truly move in space when delineating PPS [see Serino et al. (2018) for a recent evolution of the tools utilized to index PPS psychophysically].

It is worth noting that, in performing these simulations with static sounds, all model parameters and mechanisms were kept unchanged as when performing simulation with moving sounds. However, while velocity-modulation of PPS size is mediated by neural adaptation (in fact removal of neural adaptation eliminates speed effects), PPS resizing as a function of sound intensity does not rely on neural adaptation but it is the consequence of neuron’s activation function (in particular note that the role of neural adaptation is negligible here since in the first set of simulations stimuli are of short duration, and in the second set of simulation the progressive increase in sound intensity overcomes threshold increase due to adaptation).

### Neural Network Results: Simulation of Neurophysiological Data in Literature

**Fig. 17.**
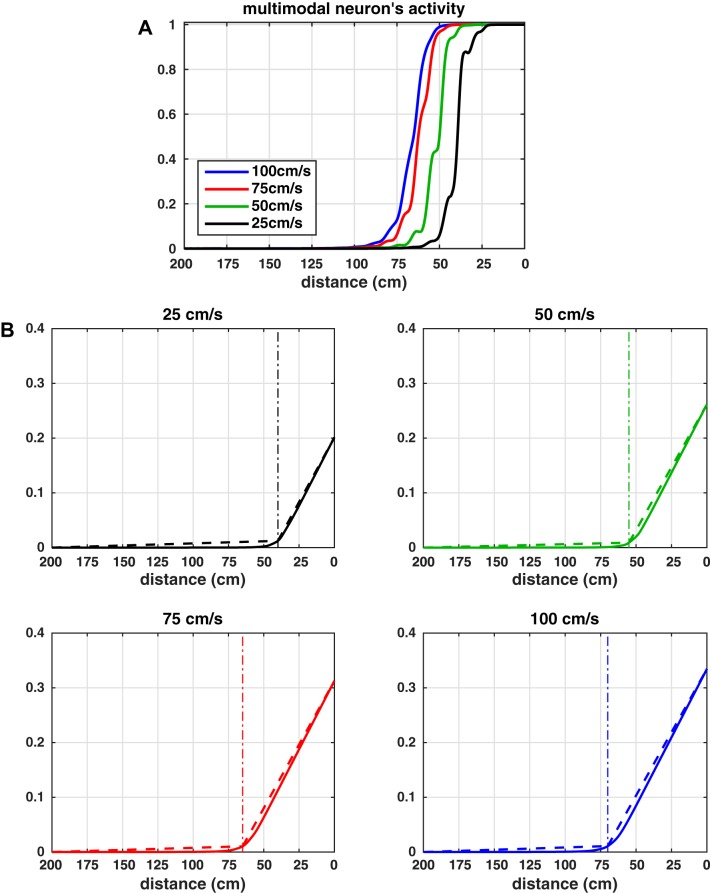
*A*: response of the multisensory neuron in the peri-face space network to a sound given alone, moving at four different velocities, as a function of the sound distance from the face. *B*: application of the procedure for estimating the size of the multisensory neuron’s auditory reaction time (RF) [according to Fogassi et al. (1996)], to the multisensory neuron responses displayed in *A*. Each plot shows the cumulative sum (normalized to the overall length of the simulation) of the multisensory neuron response to a given sound velocity and the two-segment piecewise linear function that best fitted the cumulative sum. The bending point (D*) of the piecewise linear function is taken as the size of the multisensory neuron’s auditory RF (see also methods). The same procedure is adopted for the peri-trunk space network.

In addition to accounting for the behavioral results here presented, we queried whether the model developed was able to generalize and whether its latent variables were in accord with seminal electrophysiological findings by Fogassi and colleagues (1996). Indeed, not only does the network predict faster reaction times to touch as audio stimuli move closer to the body (face or trunk) in a velocity-dependent manner, but importantly the latent variable of neural activity shows that the multisensory neuron exhibits higher levels of response at farther distances in case of fast sounds, due to the higher level of activity in the auditory area (see Fig. 17*A*, in the exemplary case of the peri-face space network, for the same four sound velocities used in the previous simulations). The sound distance at which the multisensory neuron starts to respond above baseline level was thus objectively computed (see methods; Fig. 17*B*) and taken as the size of the (simulated) multisensory neuron’s auditory RF, following the same procedure adopted in Fogassi et al. (1996); see also methods. It must be clarified that in the case of replication of behavioral results, activity in the tactile area is transformed into a RT, and this value is compared with in vivo experiments. In this case, contrarily, we utilize simulated neural activity in the multisensory area as resulting from unimodal auditory stimulation to index the transition from outside the multisensory neuron’s RF (activity no different from baseline) to inside the RF (neural activity different from baseline).

**Fig. 18.**
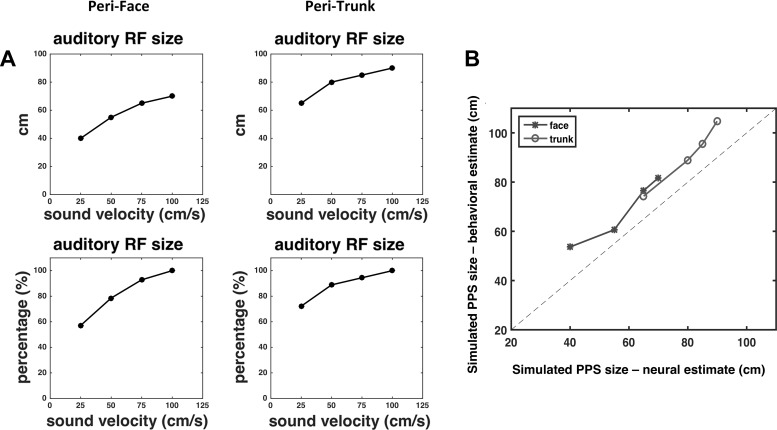
*A*: changes in the size of the multisensory neuron’s auditory reaction time (RF) as a function of sound velocity estimated with the peri-face space and peri-trunk space network, for the four velocities (25, 50, 75, 100 cm/s) used in the previous simulations. The procedure adopted for the computation of the size of the auditory RF is illustrated graphically in Fig. 13 and explained in section methods. *B*: comparison between the size of the peripersonal space (PPS) estimated with the network according to the behavioral procedure (i.e., on the basis of the multisensory facilitation in tactile RTs, *y*-axis) and the size of the PPS estimated with the network according to the neurophysiological measure (i.e., on the basis of the size of the multisensory neuron’s auditory RF, *x*-axis). The dashed line is the identity line.

According to our network results, increase in sound velocity produces an expansion in the size of the multisensory neuron’s auditory RFs, both in the peri-face space network and peri-trunk space network (Fig. 18*A*). In the range of the examined velocities 25−100 cm/s, this relationship is quasi-linear. In particular, peri-face space network results are close to those reported by Fogassi et al. (1996), with reference to the depth of the visual RF of multisensory neurons having tactile RF on the face. Similarly to our simulated data here, Fogassi et al. (1996) showed that, in the range between 20 and 80 cm/s of the visual stimulus velocity, most neurons exhibited maximal visual RF’s depth at the maximal tested velocity (80 cm/s), and that these neurons exhibited a quasi-linear decrease of RF depth as velocity decreased, reaching ~1/3–1/2 of the maximal depth at the minimum tested velocity (20 cm/s).

Beyond proving the ability of the network in reproducing neurophysiological data, we took advantage of in silico results to perform broader analyses and provide further novel predictions. First, we investigated the relationship between the PPS size estimated with the network according to the behavioral procedure (i.e., based on the simulated values of multisensory facilitation in tactile RT) and the PPS size estimated with the neural network according to the neurophysiological measure, i.e., based on the size of the multisensory neuron’s RF. As shown in Fig. 18*B* the two measures are highly correlated, both in the case of the peri-face space network and peri-trunk space network. The neural network estimates of the PPS size based on the behavioral procedures appear to exhibit a systemic positive bias of ~10–15 cm compared with the neural network based neurophysiological estimates of the PPS size. This result can be interpreted considering that the neurophysiological measure of the PPS size is obtained via computation in unisensory condition, while the behavioral proxy of PPS size is obtained via computation in the multisensory condition. Thus, the latter approximation to PPS size benefits of the enhancement and inverse effectiveness properties (Murray and Wallace 2012), which are present in multisensory but not unisensory conditions. This distinction between audio-alone and audio-tactile conditions is well illustrated in Fig. 8*A*, where the combination of the tactile input with the slow (25 cm/s) auditory input at 50 cm distance produces a disproportionate increase in the multisensory neuron response, which affects the tactile RT. Therefore, even if the sound alone traveling at 25 cm/s is able to trigger a response in the multisensory neuron only at 40 cm or less, it can nonetheless contribute to enhance the response in the tactile modality at farther distances. Therefore, the model predicts that, at a given stimulus velocity, the behavioral proxy of PPS in multisensory conditions can be larger (up to a few centimeters) compared with the neurophysiological estimate of the PPS in the unisensory condition.

### Neural Network Results: Extension of Neurophysiological Data in Literature

**Fig. 19.**
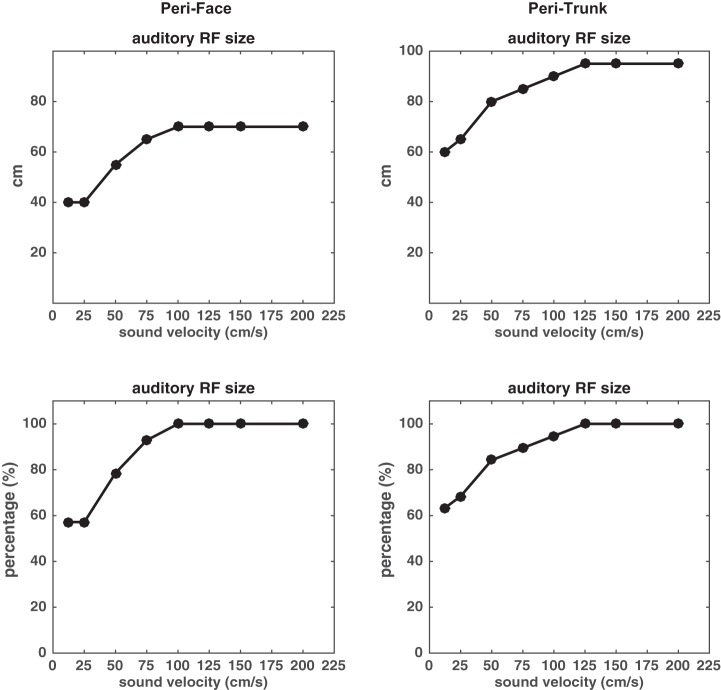
Changes in the size of the multisensory neuron’s auditory reaction time (RF) as a function of sound velocity estimated with the peri-face space network and peri-trunk space network in a wider range of velocities (from 12.5 to 200 cm/s) compared with Fig. 18. The broader analysis reveals a sigmoidal-shape relationship. The points at the velocities 25, 50, 75, 100 cm/s are the same as in Fig. 18.

We enriched the analyses by simulating additional (slower and faster) sound velocities (12.5, 125, 150, 200 cm/s) and deriving a more exhaustive picture of the relationship between the size of the multisensory neurons’ RF and sound velocity (Fig. 19). Results suggests this relationship has a sigmoidal shape, the RF’s size exhibiting no or only modest changes at velocities smaller than 25 cm/s and larger than 100–125 cm/s. Moreover, due to the correlation between RF’s size and behavioral PPS representation, the network predicts a saturation effects at behavioral level too. This sigmoidal shape can have an ecological significance. The lower saturation can ensure the activation of the PPS representation even in case of slowly approaching stimuli, which is of particular relevance for protective and defensive goals. Interestingly, this prediction is contrarily to a hypothesis generated by artificial intelligence (as opposed to biological plausibility), namely that there is an emergent nonzero cutoff speed whereby slower stimuli do not induce any prediction of touch and thus do not lead to a PPS representation (Straka and Hoffmann 2017). The upper saturation for fast moving stimuli may reflect physical limits of our sensory-motor system.

## DISCUSSION

Overall, our behavioral data seemingly indicate that the velocity of approaching sounds significantly modulates the size of audio-tactile PPS representation both around the face and the trunk. Specifically, while the size of PPS around face was ~52 cm (similar to the estimate in Serino et al. 2015b) at 25 cm/s, this changed when the velocity of incoming sounds was tripled and we here measured the boundary of peri-face space at ~77 cm. Similarly for the trunk, when auditory stimuli loomed at 25 cm/s, auditory information commenced to facilitate tactile RTs at ~83 cm (similar to Serino et al. 2015b), a distance that increased until ~103 cm when sound velocity reached 75 cm/s. This data represents the first psychophysical demonstration in humans that PPS representation is modulated as a function of stimuli velocity, as originally demonstrated at the single-cell level in nonhuman primates (Fogassi et al. 1996; see also Colby et al. 1993 and Bremmer et al. 2013). Furthermore, to the best of our knowledge, the current study is the first to suggest a remapping of PPS as a function of the velocity of incoming auditory, as opposed to visual (Bremmer et al. 2013; Colby et al. 1993; Fogassi et al. 1996) stimuli.

Importantly, in line with our previous studies (Magosso et al. 2010b; Serino et al. 2015a), we attempt to go beyond a pure observational description of PPS’s dynamism by providing a mechanistic framework via neurocomputational modeling. Indeed, while the original neurophysiological reports detailing visuo- and audio-tactile interactions as a function of depth (Duhamel et al. 1998; Fogassi et al. 1996; Graziano et al. 1997, 1999; Iriki et al. 1996; Rizzolatti et al. 1981) undoubtedly launched a field of study, they remained quasi-silent with regard the computation or underlying cellular, molecular, or systems-level processes involved in the dynamic properties of PPS (see Ishibashi et al. 2002 and Hihara et al. 2006 for plasticity properties).

Indeed, computational models are a fundamental tool to link empirical data to their underlying neurobiological processes, and hence a number of groups have made use of them to account for PPS-related phenomena. While other groups developed either theoretical (Brozzoli et al. 2012b; Makin et al. 2008) or mathematical (see, e.g., Bufacchi et al. 2016) models of PPS, we have implemented a neural network model attempting to directly link PPS properties to biologically plausible neural computations. Implementing the architectural scheme of feedforward-feedback synapses between unisensory and multisensory areas (Magosso et al. 2010a, 2010b; Serino et al. 2015a), the model has previously proved to be capable of replicating basic phenomena associated with peri-hand space, such as the enhancement of tactile detection when exteroceptive sensory signals are presented near as opposed to far from the body. Moreover, by including Hebbian rules (Hebb 1949), the model was able to replicate the seminal observation that PPS expands during tool use (Canzoneri et al. 2013; Iriki et al. 1996).

Here, the proposed neural network shares the architecture of our previous models and inherits parameter values (e.g., Magosso et al. 2010b; Serino et al. 2015a), implementing recurrent synapses between unisensory and multisensory areas; however, it equally includes two main novelties. First, the proposed model is able to account for the larger extent of the PPS around the trunk than the face, ascribing their different sizes to different synaptic patterns connecting the unisensory auditory areas to the multisensory PPS areas devoted to peri-trunk and peri-face representation. This has been obtained by changing a minimum number of parameters in the synapses (only parameters *X_C_* and *Y_C_* in *Eq. 7*, defining the extension of the region bordering the body part, see Table 1), while keeping all other parameters the same. This is significant in view of a general validity of the network architecture and parameters in describing PPS representation around the different body parts (Serino et al. 2015b). Second, the proposed model is equally able to account for the remapping of PPS as a function of sound velocity, ascribing this dynamic change to a neural adaptation to sustained stimulation. Importantly, the neural adaptation does not occur in the multisensory area (we assumed a negligible adaptation mechanism for the multisensory neurons), but in the upstream unisensory areas, as suggested in literature (Bruno et al. 2010; Elliott et al. 2009; Heron et al. 2013). Specifically, our network indicates that the sensitivity of the multisensory neuron to sound velocity is acquired through a coding mechanism of the sound velocity by the upstream auditory area, where adaptation takes place. The adaptation mechanism of the auditory neurons leads to a stronger decrease in auditory neurons’ responsiveness in case of slow-moving sounds than fast-moving sounds, so that higher level of activities of auditory population are associated to faster velocities. Crucially, the values ascribed to the adaptation mechanism must be elevated enough to allow the mechanism to play a role in overall neural activation level, but the precise values adopted are irrelevant to the replication of a velocity dependent PPS.

Adaptation, i.e., a decline in neuron’s responsiveness depending on the firing history of the neuron, has been observed at many levels in the auditory system: in the auditory nerve (Chimento and Schreiner 1991), the inferior colliculus (Gutfreund and Knudsen 2006), and the auditory cortex (Mickey and Middlebrooks 2005; Wehr and Zador 2005). It is usually described in terms of the decline in the neuron response to a second auditory stimulus, induced by a preceding one. Several mechanisms have been postulated to interpret this phenomenon including both synaptic effects (long-lasting inhibition and/or reduction in synaptic drive, Wehr and Zador 2005) and intrinsic cellular properties (change in the properties of voltage-gated calcium or potassium channels; Borisyuk et al. 2002; Gollisch and Herz 2004). Here, we aimed to maintain the network at its minimum level of complexity, and we avoided to include multiple stages of auditory processing (as it would be required for reproduction of synaptic effects) or to describe each cell in terms of its voltages and currents (as it would be required for reproduction of intrinsic cellular properties). Rather, we modeled adaptation in an empirical—although biologically reasonable—way, assuming a dynamic shift in the neuron’s activation function: decrease in responsiveness (shift of the activation function toward higher input levels) follows high and sustained neuron excitation and increase in responsiveness (shift of the activation function toward lower input levels) follows low or absence of excitation. Although we do not aspire to provide a detailed quantitative reproduction of auditory phenomena, the implemented mechanism with the given parameters can provide a qualitative agreement with neurophysiological data. First, simulated auditory neuron’s responses to stimuli of different durations resemble those observed in vivo, longer stimulation producing a progressive response’s decline (Fig. 2). Second, the network is able to reproduce the depressing effect of an excitatory priming stimulus on the response to a subsequent stimulus, even at large interstimulus interval (stimulus onset asynchrony > 500 ms, Wehr and Zador 2005, via the large value of parameter T). Finally, the network predicts a shift of the RF of the auditory neurons toward the approaching sound (see Fig. 4*B*), as observed in real auditory neurons (Wilson and O’Neill 1998; Witten et al. 2006), but unexplored within the system encoding for PPS. Interestingly, Witten et al. (2006) interpreted this effect with a mathematical model implementing a mechanism similar to ours: the response of the neuron was modulated by a negative feedback component that caused strong previous responses to decrease the gain of future responses and weak previous responses to increase the gain of future responses.

The main prediction here is that the adaptation of auditory neurons can implement the sensitivity to sound velocity, accomplished at the level of an auditory neuronal population: sound velocity is coded by the overall amount of the auditory population activity, slower velocities being associated to a lower degree of activity and faster velocities being associated to higher degree of population activity. This prediction can be tested in future experiments; however, preliminary support comes from recent studies suggesting that higher motion velocities of sound are associated to stronger cortical responses (Getzmann 2009). The multisensory neuron inherits the sensitivity to sound velocity from the auditory population via the feedforward synapses. The sensitivity of the multisensory neuron to sound velocities dictates the dynamic update of PPS representation on a trial-by-trial basis, as a function of the velocity of the auditory stimulus administered in that given trial. The distinction between the current postulation of neural adaptation as the mechanism behind the velocity-dependent mapping of PPS (i.e., a dynamic process) vs. the Hebbian learning, as the mechanism behind the expansion of PPS during tool use (i.e., a plastic process; Serino et al. 2015a) is crucial. That is, adaptation does not require repeated instances of exposure to same phenomena to demonstrate a measureable effect in firing rate, as Hebbian learning is. As such, by postulating that firing rates of neurons (auditory ones, here) involved in the PPS network decrease as a function of the duration for which they fire, we are able to account for the inherent, at single-trial level, PPS dynamicity depending on stimulus speed. The evolutionary benefit of this inherent plasticity of PPS fits well within the proposition that PPS encodes for a safety area around the body and is important for survival (Graziano and Cooke 2006). Anticipated coding of stimuli approaching the body at faster velocity might be a key property to prepare defensive responses to potential harms.

In fact, in this same vein Cléry and colleagues have demonstrated behaviorally that nonhuman primates may anticipate the spatiotemporal location of visual stimuli looming into the PPS (Cléry et al. 2015a) and that this predictive mechanism enhances tactile sensitivity. Furthermore, these researchers have recently demonstrated that the prediction of impact onto the body is accomplished via multisensory integration within the PPS (Cléry et al. 2017). The temporal prediction window or interval over which tactile sensitivity was enhanced depended on the speed of the looming stimulus (Cléry et al. 2015a). Here, thus, we postulate that this predictive mechanism may be subserved by neural adaptation within the PPS circuitry. Indeed, a time-resolved elevation in the threshold required for topographically aligned auditory neurons to drive PPS neurons may serve as a neural trace indicating the location of sounds in the immediate past. As neural adaptation depends on a neural time constant that is immutable, the length of the discernable “threshold” trace would indicate the speed of incoming stimuli. In other words, in the brain, a looming sound approaching the body leaves behind a trace of neurons that now are subject to neural adaptation. The neurons encoding for a position the sound hasn’t been at will be submitted to no neural adaptation (thresholds to evoke a response are at a baseline value), while the neurons encoding for a spatial location that the sound has been at will have a higher threshold to invoke a response (i.e., neural adaptation). Hence, the direction and length of this neural adaptation trace could theoretically inform an observer of the spatiotemporal location of a sound both in the past and in the future (assuming the sound has not changed direction or speed). In short, neural adaptation may play an important role in engendering prediction.

Interestingly, according to our neural network model, the interplay between PPS encoding and environmental predictions does not end with the potential location and timing of object-body impacts. In fact, simulations results suggest that different stimulus attributes other than speed can modulate PPS size. As presented in Fig. 13, which analyzes the effect of the looming stimuli’s intensity and spatial precision, the model suggests that while PPS size scales positively with stimuli intensity, it shrinks as the spatial location of exteroceptive stimuli becomes more precise. Similarly, these same attributes seem to modulate PPS size even in the case of static stimuli, as suggested by Figs. 15 and 16. Incidentally, PPS size modulation by stimulus speed and PPS size modulation by stimulus intensity/precision, while both rely on different levels of activation in the auditory area, are mediated by different mechanisms: speed modulation depends on the adaptation mechanism (in fact it disappears when neural adaptation is eliminated; see Fig. 11), while precision/intensity-modulation is intrinsic to auditory neurons’ activation function.

The model predictions regarding PPS size as a function of stimuli intensity and precision may be of relevance beyond the sensory domain. Indeed, arguably a number of higher-order cognitive manipulations, such as increasing or decreasing the valence attributed to a sensory stimulus via either personality traits/emotional attributes or allocation of attention may distinctly influence the perceived intensity or precision of stimuli in space (see Denison et al. 2017, for a recent demonstration of attention sharpening sensory representations). Thus, these higher-order attributes may distinctly impact PPS representation (see de Lourenco et al. 2011; Sambo and Iannetti 2013), and this may not be limited to approaching stimuli but may occur in case of stationary stimuli too (Valdés-Conroy et al. 2012). This is a novel prediction that ought to be examined in the future in case of both moving and static stimuli, to help expending our knowledge on how the nature of sensory stimuli in the environment, and our representation thereof, may play a role in shaping bodily self-consciousness and higher-order cognition more generally.

Besides interpreting mechanistically the behavioral speed-dependent modulation of PPS and providing novel predictions regarding the effect of other stimuli attributes on PPS, the network is utilized to relate our behavioral estimate of PPS to a neural measure of PPS based on multisensory neurons’ response. To this aim, we took advantage of the possibility of analyzing latent variables of the network (i.e., variables not directly accessible in human behavioral studies). Specifically, being able to simulate activity in the multisensory PPS neurons, we applied the same neurophysiological analysis as in Fogassi’s seminal paper (Fogassi et al. 1996) and computed the size of the auditory RF of the network’s multisensory neuron in response to an approaching sound traveling at different velocities. That is, as in Fogassi et al. (1996), we defined the presence of the PPS boundary (e.g., boundary of the RF) as the inflection point in the cumulative sum of spikes—this later one being a latent variable in our model, thus bridging between behavioral measures of PPS boundary (based on RT patterns) and neurobiological measures of PPS boundary (based on spike count measures). This neurophysiological-like approach was utilized to cover the same range (25–100 cm/s) mapped behaviorally (that is, on the basis of tactile RTs in multisensory condition) and further extended below (down to 12.5 cm/s) and above (up to 200 cm/s) it. Two main remarks can be drawn from these further analyses. First, the behavioral proxy of PPS size and the size of the multisensory neuron’s auditory RF (which can be considered a neurophysiological measure of PPS size) are strongly correlated, both showing a gradual and quasi-linear increase in size within the range of velocities between 25 and 100 cm/s. These network results exhibit a striking similarity to those engendered by single cell recordings in Fogassi’s 1996 paper. Second, the quasi-linear velocity-dependent modulation of PPS size appears to be restricted within the previously stated range of velocities. Indeed, the network predicts that below 25 cm/s, as well as beyond 100 cm/s, there is no further velocity modulation of PPS. This finding is reminiscent of an effect within the image speed and motion perception literature. Namely, that a static flash of light cooccurring in space and time with an approaching object is perceived as lagging behind the object (flash-lag effect, MacKay 1958). The lag is greater the faster the incoming stimuli, though the effect eventually saturates and appears to be governed by the natural statistical of spatial frequency in the world (Wojtach et al. 2008). Similarly here, within the framework proposing that the encoding of PPS is crucial in self-protective mechanisms (Graziano and Cooke 2006), we postulate that the saturation of the velocity-dependent enlargement of PPS is likely driven by the statistics of the world and evolutionary pressures, as well as biophysical constraints. These upper and lower saturation levels of the PPS velocity-dependent modulation predicted by the network may be tested in future via both behavioral and electrophysiological experiments.

In conclusion, the present study provides the first empirical evidence in humans about a key dynamic property of the PPS system, i.e., the regulation of the PPS dimension (putatively depending of the size of PPS neurons’ receptive field) as a function of the velocity of approaching stimuli. At a neurophysiological level, we postulated that a neural adaptation mechanism implemented within an architecture of recurrent connections between unisensory and multisensory areas can explain the inherent dynamism of PPS size as a function of incoming stimuli velocity. A neural network embedding this adaptation principle in its architecture to reproduce the peri-face and peri-trunk space representation was able to reproduce both the novel behavioral results obtained in humans and neurophysiological data in literature. Moreover, the correlation between RFs estimates and the behavioral proxy of PPS size in multisensory condition evidenced by the network reinforces the postulation that multisensory enhancement of tactile processing (as measure from tactile reaction times) as sounds approach the body can be taken as a psychophysically index of the extent of PPS representation and may reflect, at a population level, the RF’s depth of PPS multisensory neurons. Finally, the results of this investigation generate a number of predictions (i.e., change in PPS size as a function of sound intensity, localizability, and putatively higher order attributes of the stimuli), which can be tested in future experiments. This ability of suggesting novel hypotheses for designing further experiments, whose results would validate or correct the model, is one of the key added values of the combined empirical-modeling approach used in the present study.

## GRANTS

A. Serino and O. Blanke are supported by grants from the Swiss National Science Foundation and the Bertarelli Foundation. J. P. Noel was supported by a Fulbright fellowship via the United States Department of State Bureau of Education and Cultural Affairs and is currently supported by NIH/NIMH (1F31MH112336).

## DISCLOSURES

No conflicts of interest, financial or otherwise, are declared by the authors.

## AUTHOR CONTRIBUTIONS

J.-P.N., O.B., and A.S. conceived and designed research; J.-P.N. performed experiments; J.-P.N. and E.M. analyzed data; J.-P.N., O.B., E.M., and A.S. interpreted results of experiments; J.-P.N. and E.M. prepared figures; J.-P.N., E.M., and A.S. drafted manuscript; J.-P.N., O.B., E.M., and A.S. edited and revised manuscript; J.-P.N., O.B., E.M., and A.S. approved final version of manuscript.
